# Minimizing patients total clinical condition deterioration in operating theatre departments

**DOI:** 10.1007/s10479-022-05046-y

**Published:** 2022-11-18

**Authors:** Omolbanin Mashkani, Andreas T. Ernst, Dhananjay Thiruvady, Hanyu Gu

**Affiliations:** 1 School of Mathematical and Physical Sciences, University of Technology Sydney, Ultimo, Sydney, NSW 2000 Australia; 2 School of Mathematics, Monash University, Clayton, Melbourne, VIC 3800 Australia; 3 School of Information Technology, Deakin University, Waurn Ponds, Geelong, VIC 3216 Australia

**Keywords:** Operating theatre, Master surgical schedule, Surgical case assignment, Clinical condition deterioration

## Abstract

The operating theatre is the most crucial and costly department in a hospital due to its expensive resources and high patient admission rate. Efficiently allocating operating theatre resources to patients provides hospital management with better utilization and patient flow. In this paper, we tackle both tactical and operational planning over short-term to medium-term horizons. The main goal is to determine an allocation of blocks of time on each day to surgical specialties while also assigning each patient a day and an operating room for surgery. To create a balance between improving patients welfare and satisfying the expectations of hospital administrators, we propose six novel deterioration rates to evaluate patients total clinical condition deterioration. Each deterioration rate is defined as a function of the clinical priorities of patients, their waiting times, and their due dates. To optimize the objective functions, we present mixed integer programming (MIP) models and two dynamic programming based heuristics. Computational experiments have been conducted on a novel well-designed and carefully chosen benchmark dataset, which simulates realistic-sized instances. The results demonstrate the capability of the MIP models in finding excellent solutions (maximum average gap of 4.71% across all instances and objective functions), though, requiring large run-times. The heuristic algorithms provide a time-efficient alternative, where high quality solutions can be found in under a minute. We also analyse each objective function’s ability in generating high quality solutions from different perspectives such as patients waiting times, the number of scheduled patients, and operating rooms utilization rates. We provide managerial insights to the decision makers in cases where their intention is to meet KPIs and/or maintaining trade-offs between patients and administrators expectations, more fair assignments, or ensuring that the most urgent patients are taken care of first.

## Introduction

Hospitals are typically a major health facility in a region. The operating theatre (OT) department within hospitals typically consists of several operating rooms (ORs), and they are the most critical and costly department because of their operational complexity as well as the scarceness and high costs of resources (Guerriero and Guido, 2011). Among surgeries in a hospital, approximately 60–70% are surgical cases and the OT itself accounts for more than 40% of the hospitals total expenses (Denton et al., 2007). This underlines the necessity for developing efficient OT operations management approaches in order to reconcile supply with demand (planning) and making time-related decisions (scheduling) (Guerriero and Guido, 2011; Samudra et al., 2016). The OT planning and scheduling process is highly complicated due to different stakeholder interests and preferences, and involves three hierarchical stages including strategical, tactical, and operational stages (Rachuba and Werners, 2017). The hierarchical stages are entirely dependent on each other and decisions made in each stage can significantly affect the subsequent stages (Guerriero and Guido, 2011).

Strategic planning (known as the case mix planning problem (CMPP) deals with making long-term decisions on the total OT capacity that can be assigned to each surgical group (so-called surgical specialty) and the ideal composition and volume of patients that can be operated upon (Wagner, 1975). The second stage is tactical planning which follows the hospital’s strategic decisions to determine the allocation of OT time to specialties while still not considering individual patients (Koppka et al., 2018). At this stage, the capacity of ORs in terms of time blocks is shared between surgeons/surgical specialties to generate a cyclic timetable, known as master surgical schedule problem (MSSP), over a medium-term planning horizon (Beliën and Demeulemeester, 2008). The operational stage is mainly concerned with assigning patients to ORs and making time-related decisions (Koppka et al., 2018). The operational planning problem is split into two main sub-problems including the surgical case assignment problem (SCAP) and the surgical case sequencing problem (SCSP). While the SCAP assigns patients to ORs and determines a particular surgery day and OR for each patient, the SCSP focuses on timing aspects and sequencing the patients in the ORs (Aringhieri et al., 2015).

The COVID-19 pandemic has resulted in elective surgeries being put on hold repeatedly during lock-downs in Australia and in other countries around the world. This has resulted in long waiting lists that need to be processed (Arab Momeni et al., 2022). To reduce these waiting lists, it is essential to make the best use of the scarce resources available to hospitals, particularly the OT resources. This paper considers the combined problem of developing master surgical scheduling (MSS) at tactical stage and the operational case assignment problem, namely MSS-SCAP, in order to produce more efficient schedules. The ultimate goal of the MSS-SCAP is to create a timetable of surgical specialties allocation to time blocks and identify every patient a particular surgery day and OR.

When hospital administrations assign time blocks to surgical specialties, they typically consider criteria based on equity and fairness, which leads to priorities being assigned to patients (Samudra et al., 2016). When patients are assigned to ORs, those with higher priorities (most critical clinical conditions) will be operated on as early as possible.In order to cope with the MSS-SCAP challenges, improve patients welfare and satisfy administrators expectations, we quantify the deterioration of patients clinical condition by introducing six new deterioration rates, each as a function of patients clinical priorities, waiting times, and their due dates. We further discuss each deterioration rate’s capability in providing high quality solutions in terms of fair patient assignment, lower waiting times, larger number of accepted patients, providing timely care to most urgent patients, and larger OT utilization rates. We note that while some of these quality measures could be incorporated directly in the optimisation, most of them are about the longer term effectiveness over multiple planning periods. To test this, we examine the outcomes when the objectives are employed in a rolling horizon method.

Beliën and Demeulemeester proved that the MSSP is an NP-hard problem. They achieved this by reduction from 3-Partition problem which is a generic version of the classic bin-packing problem (Beliën and Demeulemeester, 2004) (see also Tànfani and Testi (2010)). Freeman et al. showed that the SCAP is an NP-hard problem through a reduction of traveling salesman problem (Freeman et al., 2016). Since both MSSP and SCAP are NP-hard problems, therefore their integrated problem, i.e. MSS-SCAP, is also an NP-hard problem (see Aringhieri et al. (2015)).

To hedge against the complexity of the MSS-SCAP and minimize patients total clinical condition deterioration, we develop a MIP model and two dynamic programming based heuristics, called DPH1 and DPH2. The MIP model provides a framework to quantify the solution quality while the heuristic algorithms aim to find high quality solutions in short time-frames. While there exists a large body of literature on OT planning and scheduling problems, most of them have carried out their computational studies based on real-world case studies and hence, the disclosure of their datasets is almost nonexistent (largely due to data confidentiality). Thus, another novel aspect of this study is to develop a comprehensive dataset (inspired by previous studies), which provides real-size instances that serve as a benchmark and can be utilized by researchers in future for their computational studies. The major contributions of our study can be summarized as follows:

- Proposing novel objective functions to ensure effective OT scheduling, by penalising the patients’ potential clinical condition deterioration.
- Introducing a well-structured benchmark dataset to the domain of OT planning and scheduling problems.
- Presenting a novel MIP model and two dynamic based heuristics to minimize patients’ total clinical condition deterioration.

The remainder of this paper is organised as follows. In Sect. 2, we discuss the relevant literature in the OT planning and scheduling domain. In Sect. 3, problem definition, assumptions, and the MIP model are provided. Section 4 reports splitting the main problem into several knapsack sub-problems, which are handled by the two dynamic programming based heuristics. In Sect. 5, the benchmark instances are discussed and computational results of running the MIP model and heuristic algorithms on the benchmark instances are provided. Finally, Sect. 6 concludes the paper and provides suggestions for future research.

## Literature review

In the past two decades, many researchers have investigated the OT planning and scheduling problems. Thorough surveys on the OT planning and scheduling literature and its challenges have been provided in Guerriero and Guido (2011), Samudra et al. (2016), May et al. (2011), Hof et al. (2017), Zhu et al. (2019). Intending to decrease the complexity and better characterization of the overall problem, much of literature has studied just one planning stage at a time. However, investigating multiple stages simultaneously may result in having more integrated, stable and efficient planning that is more likely to succeed (Hashemi et al., 2016). In this section, we first review the related literature addressing more than one stage of the planning process, in Sect. 2.1. Then in Sect. 2.2, we review the literature focusing on patients prioritization and their clinical condition deterioration. At the end, we discuss our motivation and contributions to the literature.

### The integrated problems

Despite the strong dependency between the three planning stages, much of the literature has only studied a single stage (Guerriero and Guido, 2011). Testi et al. dealt with the three stages of the OT planning and scheduling problem by developing a three-phase approach (Testi et al., 2007). First, they developed an integer linear programming (ILP) model to solve the CMPP and then taking the results as resource constraints, they generated a weekly MSS. Given the MSS, a simulation model is suggested to address SCAP and SCSP at the third phase. A few researchers have considered both MSSP and SCAP concurrently to address the tactical and operational planning problems. Testi et al. also investigated the MSS-SCAP and proposed an ILP model and an algorithm for patients prioritization and assigning them to the ORs (Testi and Tànfani, 2008). To handle the same problem, Tanfàni et al. developed an ILP model and a three-step heuristic algorithm. Several constraints contributed to the complexity of their study such as expected patients length of stays, resource availability, and hospital budget constraints (Tànfani and Testi, 2010).

Adan et al. also studied the MSS-SCAP and provided a MIP model and a genetic algorithm to plan surgeries and specialties taking into account several resource constraints such as the OT capacity, nursing hours, and the capacity of downstream resources (Adan et al., 2011). Agnetis et al. discussed the effect of allowing MSS flexibility on the quality of the surgical plans and assignment of patients to ORs (Agnetis et al., 2012). First they propose an ILP model to treat patients within their advised due dates over a one-week planning horizon. Next, system behaviour is simulated over one year planning horizon. They extended their study and proposed a two-phase decomposition approach to handle the MSS-SCAP (Agnetis et al., 2014). Aringhieri et al. also investigated the MSS-SCAP and suggested a MIP model and a hybrid metaheuristic algorithm combining tabu search, local search, and a few greedy constructive algorithms (Aringhieri et al., 2015).

In addition, Spratt and Kozan suggested a mixed integer nonlinear programming model and combined simulated annealing and reduced variable neighborhood search to address the MSS-SCAP (Spratt and Kozan, 2016). They formulated the strategic decisions and uncertainty of surgery durations as model constraints. In Guido and Conforti (2017), authors proposed a hybrid genetic algorithm to create an MSS and assign patients to days and time blocks. Anjomshoa et al. studied the MSS-SCAP and developed a MIP model to optimize a multi-criteria objective function (Anjomshoa et al., 2018). Mashkani et al. also investigated the MSS-SCAP aiming to minimize the total patients welfare loss resulted by their excessive waiting times and proposed a MIP model, two heuristic algorithms and one iterated local search. They interpreted the assignment of patients to each time block as single knapsack problems and solved them using dynamic programming. The heuristic solutions are used as initial solution for the iterated local search (Mashkani et al., 2020).

The MSSP, SCAP, and SCSP are investigated all-together in Roshanaei et al. (2017) where patients, surgeons, and ORs are collaboratively shared amongst a coalition of hospitals. The authors proposed a mixed-integer dual resource constrained model as well as a logic-based benders decomposition, which decomposes the overall problem into an allocation master problem and sequencing sub-problems. Furthermore, a game theoretic approach is proposed to ensure the fairness of collaborative schedules. Burdett and Kozan studied improving patients flow from admission to discharge in one integrated problem of MSSP, SCAP, and SCSP (Burdett and Kozan, 2018). They model the problem as a flexible job-shop scheduling problem with blocking and no-wait constraints which patients, beds, wards and treatment activities are considered as jobs, single machines, parallel machines and operations, respectively. To solve the problem, constructive algorithms and hybrid metaheuristics are developed.

Arab Momeni et al. investigated the MSS-SCAP to assign operating rooms to the COVID-19 and non-COVID-19 patients during pandemic of the Coronavirus in a cardiovascular department (Arab Momeni et al., 2022). They developed a MIP model and a stochastic robust optimization approach taking into account the uncertainty of elective and emergency patients surgery durations. They analysed the MIP model for different values of the robust parameters and concluded that hospital managers could select a solution to better balance the cost, constraints violation levels as well as utilization rates of hospital facilities based on their risk-averse level.

### Patients prioritization

Prioritizing patients is highly complicated in that it determines the position of a patient on the waiting list against other patients and the severity of their clinical condition (Abbasgholizadeh Rahimi et al., 2016). Excessive patients waiting times may lead to negative consequences and deterioration of their clinical conditions. Despite the importance of patients prioritization in the planning and scheduling process, only a few researchers have taken this criteria into consideration. In 2002, the Italian Ministry of Health defined five urgency-related groups (URGs) associated with patients recommended maximum time before treatment (MTBT) and based on two criteria including (a) progression of disease; and (b) level of pain, dysfunction or disability. The five URGs have been used later in other health care systems such as Australia to prioritize patients on the waiting list (Testi et al., 2007; Valente et al., 2009).

In Testi et al. (2007), a performance criteria, known as need-adjusted-waiting day (NAWD), is introduced based on patients waiting times and MTBTs to measure the efficiency and equity of patients schedules. The NAWD evolves equally for all the patients in the same urgency category. Testi et al. proposed an objective function to quantify patients total welfare loss or in the other words patients total clinical condition deterioration (Testi and Tànfani, 2008). The deterioration is defined as a linear function of patients waiting time and priorities. This function later has been used by Aringhieri et al. (2015), Tànfani and Testi (2010) and Agnetis et al. (2014) to minimize patients total clinical condition deterioration. Min and Yih also minimized the cost of postponing surgeries using a static priority score recommended by clinical physicians at the time of patient admission (Min and Yih, 2010). Guido et al. focused on maximizing the OT utilization and the total priorities of scheduled patients at the same time (Guido and Conforti, 2017). Duràn et al. prioritized patients based on their NAWD (defined in Testi et al. (2007)) to take patients urgency and priority into account while maximizing the OT utilization (Duràn et al., 2017). Furthermore, Rahimi et al. reviewed the patients prioritization systems and proposed an assessment framework considering delay in patients treatment, risk criteria, and a profile matrix to reflect evolving patients clinical condition (Abbasgholizadeh Rahimi et al., 2016). The proposed framework helps with prioritization but not with the planning and scheduling process.

Oliveira et al. also proposed a patients prioritization system to optimize an aggregate function of maximizing total priority of assigned patients, surgeons preferences, and the ORs utilization (Oliveira et al., 2020). Mashkani et al. considered the importance of patients due dates into the clinical condition deterioration to make sure patients with severe health condition will get on time treatment and create a trade-off between the cost of accepting a surgery or postponing it Mashkani et al. (2020). If a patient is not scheduled in the current planning horizon, i.e. patient is not accepted, they will be either postponed to be treated in the next planning horizons or if their clinical condition is severe, they must be transferred to another hospital to get the required treatment in a timely fashion.

Doulabi and Pourazari considered weekly operating room planning problem with an exponential number of scenarios to minimize the sum of the fixed opening cost of operating rooms and the expected overtime costs taking into account the due dates of patients (Hashemi Doulabi and Khalilpourazari, 2022). Although they considered patient due dates, their model does not consider patient priorities for the order in which patients are treated. All patients are assumed to be scheduled, potentially incurring significant amounts of overtime.

### Motivation and contributions

As discussed, despite the strong dependency between different stages of the OT planning and scheduling process, much of the literature has only studied a single stage (Guerriero and Guido, 2011). Some studies have considered more than one stage at a time, though the proposed methodologies are not universal and generalizable to real-world surgical departments without extensive customization (Samudra et al., 2016). Nonetheless investigating multiple stages simultaneously may result in having more integrated, stable, and efficient schedules that are more likely to succeed (Hashemi et al., 2016). The main goal of this study is to provide thorough solution approaches to generate an MSS (which allocates specialties to time blocks) and cope with the SCAP (which assigns each patients to a day and a time block) all-at-once.

In spite of the efforts in the literature, the current patient prioritization tools overlook the importance of patients due dates and the impact of excessive delay on the clinical condition of patients. The prioritization should provide sufficiently robust rankings to hedge against the dynamic nature of their health condition overtime. In this study, we propose different deterioration rates to address the above-mentioned drawbacks, balance the advantages of scheduling patients based on their priority, their risk of not being accepted and their risk of surgery postponement. We extend the work of Mashkani et al. (2020) by presenting more sophisticated formulations for patients ranking and quantifying their health condition overtime. In the work of Mashkani et al. (2020) the welfare loss of patients is defined as a linear function of patients priority and their waiting times (inspired from Testi and Tànfani (2008), Tànfani and Testi (2010), Aringhieri et al. (2015)). Here, the deterioration rates are defined based on available information of patient waiting times, due dates, and their clinical priorities at the time of admission. The six rates can be beneficial from different aspects such as (a) measuring the impacts of acceptance, postponement, or rejection of patients based on their health condition; and (b) providing decision-making tools to assess the quality and efficiency of patients assignment to the ORs from different stakeholders perspectives.

Table 1 summarizes the literature related to this study. In this table, the columns *OTKPIs* show the objective functions focusing on the OT performance criteria such as OT related costs and utilization. The columns *PatientsKPIs* illustrate the studies focused on criteria related to patients. As illustrated in Table 1, just a few studies have focused on the clinical condition deterioration of patients, and solving the OT planning and scheduling problems at both tactical and operational levels. This highlights negligence of patients welfare in hospital administration and OT management decision making process.

**Table 1:** The related literature on the MSS-SCAP with their performance criteria

Paper	Problems	OT KPIs		Patients’ KPIs				
		Related costs	Utilization	Waiting times	Planned patients	Tardiness Earliness	Urgency	Welfare loss
Testi et al. (2007)	CMPP MSSP SCAP SCSP		$\checkmark$	$\checkmark$				
Testi and Tànfani (2008)	MSSP SCAP							$\checkmark$
Tànfani and Testi (2010)	MSSP SCAP							$\checkmark$
Min and Yih (2010)	SCAP						$\checkmark$	
Adan et al. (2011)	MSSP SCAP		$\checkmark$					
Agnetis et al. (2012)	MSSP SCAP		$\checkmark$		$\checkmark$			
Agnetis et al. (2014)	MSSP SCAP				$\checkmark$			
Aringhieri et al. (2015)	MSSP SCAP							$\checkmark$
Spratt and Kozan (2016)	MSSP SCAP				$\checkmark$			
Duràn et al. (2017)	SCAP		$\checkmark$				$\checkmark$	
Guido and Conforti (2017)	CMPP MSSP SCAP		$\checkmark$				$\checkmark$	
Roshanaei et al. (2017)	MSSP SCAP SCSP	$\checkmark$			$\checkmark$			
Burdett and Kozan (2018)	MSSP SCAP SCSP							
Anjomshoa et al. (2018)	MSSP SCAP				$\checkmark$	$\checkmark$		
Mashkani et al. (2020)	MSSP SCAP							$\checkmark$
Oliveira et al. (2020)	SCAP						$\checkmark$	
Arab Momeni et al. (2022)	MSSP SCAP	$\checkmark$	$\checkmark$					
Hashemi Doulabi and Khalilpourazari (2022)	SCAP	$\checkmark$	$\checkmark$					

Another motivation for conducting this study is to fill the gap of a well-designed and realistic-size benchmark dataset for OT planning and scheduling problems. Due to the confidentiality of patients health records, the disclosure of hospital data is almost impossible. Indeed, there is almost no open access dataset that could reflect the real hospital settings and be a basis for researchers to compare their methodologies (Mashkani et al., 2020). Hence, we propose a comprehensive and well-structured benchmark with 1080 instances motivated from the literature such as Testi et al. (2007), Fei et al. (2010), Spratt and Kozan (2016), which also provides other researchers with a dataset to perform their computational experiments. The benchmark suggests input values for the main parameters in a general OT planning and scheduling problem such as the scale of a typical OT department, length of planning horizon, number of surgical specialties, and patients characteristics including their MTBTs, priority scores, and waiting times. This benchmark dataset is used to thoroughly analyse our proposed solution methodologies in this study.

## Problem statement and MIP model

In the MSS-SCAP, there is a set of *s* specialties, $s \in \{1,\dots ,S\}$, to perform surgeries of *P* elective patients. Each specialty *s* has a set of $P_{s}$ patients, where $P=P_{1} \bigcup P_{2} \bigcup ... \bigcup P_{S}$ and for any *s* and $s'$, $P_{s} \bigcap P_{s'}=\emptyset$ meaning each patient *p* belongs to exactly one specialty. The surgery duration of patient *p* is denoted by $L_{p}$ which is measured in minutes. All patients release dates are at the beginning of the horizon, i.e. equal to zero. All surgeries of each specialty can be performed by any surgeon of that specialty and all ORs are sufficiently well-equipped thus usable by any specialty. The planning horizon consists of *D* days, indexed by $d \in \{1,2,\dots ,D\}$. On each day *d*, a set of *T* operating rooms are accessible, where $t \in \{1,2,\dots ,T\}$. Each OR capacity is divided into several time blocks $b \in \{1,2,\dots ,B\}$. The length of each time block *b* in day *d* is given and denoted by $\tau _{(d,b)}$ and the total processing time of the patients assigned to a time block cannot exceed it’s capacity. To assign patients and specialties to time blocks, we apply a block scheduling policy, where each time block *b* is assigned to at most one specialty and cannot be shared amongst different specialties.

Each patient *p* is given a maximum time before treatment $MTBT_{p}$ based on their URG. The MTBTs are used to recommend each patient *p* a priority score $pr_{p}$ indicating severity of their clinical condition at the time of their admission (Testi et al., 2007; Testi and Tànfani, 2008). Hence, a patient’s priority score is an indicator of how their health condition has already deteriorated and their urgency for an early surgery. At the beginning of the planning horizon, it is known that each patient *p* has already spent $W_{p}$ days on the waiting list and their surgery will be due in $Dl_{p}$ days, where $Dl_{p} = MTBT_{p} - W_{p}$.

The available capacity of all ORs over the planning horizon is generally insufficient, which means that it will not be possible to schedule all patients. Hence, a patient *p* is either to be operated on or rejected/transferred to another hospital. If patient *p* with due date within the planning horizon ($Dl_p \le D$) cannot be scheduled in time, they will be transferred to another hospital for timely treatment.

Although a patient’s clinical condition is dynamic and changes over time, in this study we consider single snapshot of time with the aim of devising plans based on a patient’s current status. We introduce six different deterioration rates (denoted by $R_{(i)}(p,d)$, $i \in {\mathcal {O}}= \{1,..., 6\}$), each as a function of patients waiting times, priority scores, due dates, and the day of surgery to measure their clinical condition deterioration. These functions take day *d* as input variable and $pr_{p}$, $MTBT_{p}$, $W_{p}$ are given parameters. When patient *p* with waiting time $W_{p}$ and due date $Dl_{p}$ is accepted for surgery on day *d*, then their clinical condition deteriorates at most by $d+W_{p}$ days. However, if patient *p* is rejected, then their clinical condition deteriorates depending on their due date. If the rejected patient’s due date is within the planning horizon ($Dl_{p} \le D$), then the patient must be transferred to another hospital to get on time treatment and their condition worsens by at least $Dl_{p}+1$ days. If the rejected patient’s due date is beyond the planning horizon ($Dl_{p} > D$), then they have to wait for treatment in the next planning horizons and their clinical condition gets worse at least by $D+1$ days. The six deterioration rates are shown in Eqs. (1) to (6).

1 $$\begin{aligned} R_{(1)}(p,d)= & {} pr_{p}\ (d+W_{p}) \end{aligned}$$

2 $$\begin{aligned} R_{(2)}(p,d)= & {} pr_{p}\ \alpha _p\, d \end{aligned}$$

3 $$\begin{aligned} R_{(3)}(p,d)= & {} pr_{p}\ \alpha _p\ \beta _p\ d \end{aligned}$$

4 $$\begin{aligned} R_{(4)}(p,d)= & {} pr_{p}\ \frac{W_{p}+d}{MTBT_{p}}\ d \end{aligned}$$

5 $$\begin{aligned} R_{(5)}(p,d)= & {} pr_{p}\ \beta _p\ \frac{W_{p}+d}{MTBT_{p}}\,d \end{aligned}$$

6 $$\begin{aligned} R_{(6)}(p,d)= & {} pr_{p}\ \frac{W_{p}+d}{MTBT_{p}} \ \frac{d}{MTBT_{p}-(W_{p}+d)+1} \end{aligned}$$

We have adapted $R_{(1)}(p,d)$, formulated in Eq. (1) from the literature (Testi and Tànfani, 2008; Tànfani and Testi, 2010; Aringhieri et al., 2015) and extended the context to investigate the cost of rejection/postponement of high priority patients on the quality of schedules and their welfare.

The Eq. (2) shows our second deterioration rate, i.e. $R_{(2)}(p,d)$. To develop $R_{(2)}(p,d)$, we incorporated the delay ratio $\alpha _p$ into $R_{(1)}(p,d)$ in order to quantify the impacts of surgery delay in patients clinical condition where $\alpha _p=\frac{W_{p}}{MTBT_{p}}$.

The third deterioration rate is illustrated in Eq. (3). In $R_{(3)}(p,d)$, we added the risk of rejection ($\beta _p$) to $R_{(2)}(p,d)$, where $\beta _p=\frac{1}{MTBT_{p}-W_{p}+1}$ and measures the impacts of the rejection probability on patients health.

It is then expected that $R_{(1)}(p,d)$ to $R_{(3)}(p,d)$ schedule high priority patients first irrespective of their due dates since patients health condition has a linear relationship with their priority scores and their actual waiting times on the list, i.e $(d+W_{p})$. However, When the planning horizon is long, patients clinical condition may get worse exponentially. Therefore, the deterioration rate will have a non-linear relationship with patients’ actual waiting times on the waiting list.

In Eq. (4), we introduced a new deterioration rate, $R_{(4)}(p,d)$, where a patient’s health condition deterioration is a simple quadratic function of their surgery day *d*. Then in Eq. (5), we incorporated a scaling factor $\beta _p$ to $R_{(4)}(p,d)$ and developed another rate as $R_{(5)}(p,d) = \beta _p R_{(4)}(p,d)$. The scaling factor is designed in such a way that $R_{(5)}(p,Dl_{p})=pr_{p}$ for any patient *p* which $Dl_{p}= MTBT_{p}-W_{p}+1$. This implies that patient *p*’s deterioration rate is equal to their priority score if their due date is within the planning horizon. Finally, $R_{(6)}(p,d)$ in Eq. (6) is defined as $R_{(6)}(p,Dl_{p})=R_{(4)}(p,Dl_{p})$ with extreme growth in the cost (penalty) as the day of surgery approaches $Dl_{p}$.

For each deterioration rate, we define an objective function $OF_{(i)}$, $i \in {\mathcal {O}}$ to measure the total clinical condition deterioration cost of all patients at the end of the planning horizon. In objective function $OF_{(i)}$, each patient *p*’s clinical condition deteriorates by $R_{(i)}(p,d)$ and the objective function calculates the total patients welfare loss at the end of the planning horizon. To have a clear understanding of how each objective function works, the first objective function is discussed here. In $OF_{(1)}$, the clinical condition of patient *p* who is to be operated on day *d* and block *b* deteriorates by $pr_{p}(d+W_{p})$. If patient *p* is not scheduled for surgery, then (a) if $Dl_{p} \le D$, they will be transferred to another hospital to get on-time treatment and their condition will deteriorate at least by $pr_{p}(Dl_{p}+1+W_{p})$; (b) otherwise they need to wait at least another $D+1$ day to get treatment and their condition will get worse at least by $pr_{p}(D+1+W_{p})$. The MIP model for $OF_{(i)}$ is presented in equations (7aa) to (7f). In this model, four sets of decision variables are used including:

- $X_{tsdb}$, a binary variable which is equal to 1 if time block *b* of operation room *t* is assigned to specialty *s* on day *d*.
- $Y_{tpdb}$, a binary variable which is equal to 1 if time block *b* of operation room *t* is assigned to perform surgery of patient *p* on day *d*.
- $U_{p}$, a binary variable which is equal to 1 if patient *p* with due date within the planning horizon ($Dl_{p} \le D$) being transformed to another hospital for on-time treatment.
- $Z_{p}$, a binary variable which is equal to 1 if patient *p* with due date beyond the planning horizon ($Dl_{p} > D$) needs to wait at least another $D+1$ days for surgery.

7a $$\begin{aligned}{} & {} \mathop {\textrm{min}}\limits _{<} \quad b\sum _{t}\sum _{p}\sum _{d\le Dl_p}\sum _{b}R_{(i)}(p,d)Y_{tpdb} +\sum _{p\mid Dl_{p} \le D}R_{(i)}(p,Dl_{p}+1)U_{p} \nonumber \\{} & {} \qquad \quad +\sum _{p\mid Dl_{p} > D}R_{(i)}(p,D+1) Z_{p} \end{aligned}$$

7b $$\begin{aligned}{} & {} \mathrm{s.t.}\quad \sum _{t}\sum _{d \le Dl_{p}}\sum _{b}Y_{tpdb}+U_{p}=1 \qquad \forall \ p\mid Dl_{p} \le D \end{aligned}$$

7c $$\begin{aligned}{} & {} \qquad \quad \sum _{t}\sum _{d}\sum _{b}Y_{tpdb}+Z_{p}=1 \qquad \quad \forall \ p\mid Dl_{p} > D \end{aligned}$$

7d $$\begin{aligned}{} & {} \quad \qquad \sum _{p \in P_{s}}L_{p} Y_{tpdb}\le \tau _{(d,b)}X_{tsdb} \qquad \qquad \forall \ t, s, d, b \end{aligned}$$

7e $$\begin{aligned}{} & {} \quad \qquad \sum _{s} X_{tsdb}\le 1 \qquad \qquad \qquad \qquad \forall \ t, d, b \end{aligned}$$

7f $$\begin{aligned}{} & {} \quad \qquad X_{tsdb},Y_{tpdb}, Z_{p}, U_{p} \in \{0,1\} \qquad \forall \ s, t, p, d, b \end{aligned}$$

Objective (7aa) minimizes the total clinical condition deterioration of all accepted, transferred, and rejected patients where the deterioration rate of patient *p* assigned to block *b* of operating room *t* on day *d* is $R_{(i)}(p,d)$ for $i \in {\mathcal {O}}$. Constraints (7b) require if the due date of patient *p* is within the planning horizon ($Dl_{p} \le D$), they must be treated within their due date or transferred to another hospital for timely treatment. Constraints (7c) ensure that a patient can be accepted at most once or rejected to be considered in the next planning horizons if their due date is beyond *D*, i.e $Dl_{p} > D$. Constraints (7d) guarantee that a patient is assigned to a time block *b* if and only if block *b* is assigned to the patient’s specialty and the total processing time of all patients assigned to block *b* must not exceed the available capacity of the block. Constraints (7e) ensure that time blocks are not shared between different specialties as per the block scheduling policy.

In this study, it is assumed that a hospital has one central OT department with several identical ORs. There is no dedicated OR with specialized equipment, so all the available ORs can be used to perform all types of surgeries. Hence, we do not consider the index *t* as part of our equations and calculations neither in development of solution methodologies, benchmark design, nor in computational experiments. However, additional or specialized equipment may restrict the amount of time that can be assigned to surgical specialties and thereby require the adaption of “suitability of the ORs constraints” into the problem, e.g. Constraints set (8) from Spratt and Kozan (2016), which can easily be adapted by the benchmark set and solution methodologies proposed in this paper.

## Solution method

In this section, we discuss our proposed dynamic programming based heuristics which we refer to as DPH1 and DPH2, respectively. For the purposes of these heuristics, we convert the objectives to maximization rather than minimization in a straightforward manner.

All the six objective functions aim to minimize the total clinical condition deterioration of all accepted, transferred, and rejected patients on the waiting list (patients welfare loss). From constraints (7b) and (7c), we replace the two decision variables $U_{p}$ and $Z_{p}$ by $1-\sum _{d\le Dl_p}\sum _{b}Y_{pdb}$ and $1-\sum _{d}\sum _{b}Y_{pdb}$, respectively. For instance, the first objective function $OF_{(1)}$ is re-written as:

8 $$\begin{aligned} OF_{(1)}&= min \sum _{p\mid Dl_{p} \le D}\sum _{d} \sum _{b} pr_{p}(Dl_{p}+1-d)Y_{pdb} \nonumber \\&\quad + \sum _{p \mid Dl_{p}> D}\sum _{d}\sum _{b}pr_{p}(D+1-d)Y_{pdb}\nonumber \\&\quad - \sum _{p\mid Dl_{p} \le D}pr_{p}(Dl_{p}+1+W_{p}) -\sum _{p\mid Dl_{p} > D} pr_{p}(D+1+W_{p}) \end{aligned}$$

Here $\sum _{p \mid Dl_{p} \le D}pr_{p}(Dl_{p}+1+W_{p})$ is a constant and represents the worst case scenario where no patient with due date within the planning horizon ($Dl_{p} \le D$) is accepted. The $\sum _{p \mid Dl_{p} > D} pr_{p}(D+1+W_{p})$ is also a constant value demonstrating the worst case scenario where all patients with $Dl_{p} > D$ get rejected. In the next step, we update the model to maximize the objective function by multiplying the objective function by $(-1)$. After eliminating the constant values, we get a maximizing function denoted by $OF^{*}_{(1)}$:

9 $$\begin{aligned} OF^{*}_{(1)}&= max \sum _{p\mid Dl_{p} \le D}\sum _{d} \sum _{b} pr_{p}(Dl_{p}+1-d)Y_{pdb} \nonumber \\&\quad + \sum _{p \mid Dl_{p} > D}\sum _{d}\sum _{b}pr_{p}(D+1-d)Y_{pdb} \end{aligned}$$

From (9), we can conclude that the deterioration rate of patient *p* on day *d* is $pr_{p}(Dl_{p}+1-d)$ if $Dl_{p} \le D$ and $pr_{p}(D+1-d)$ otherwise. By applying the same argument for the other objective functions, we will have six maximization objective functions, which are called $OF^{*}_{(i)}$ for $i \in {\mathcal {O}}$ and the deterioration rate of any patient *p* on day *d* is called $R^{*}_{(i)}(p,d)$. The $R^{*}_{(i)}(p,d)$ for all the six objectives are shown in equations (10) to (15), where $\theta _p=\min \{Dl_p, D\}+1$ and $\gamma _p = \frac{Pr_p}{MTBT_p}$.

10 $$\begin{aligned} R^{*}_{(1)}(p,d)= & {} pr_{p}\,(\theta _p-d) \end{aligned}$$

11 $$\begin{aligned} R^{*}_{(2)}(p,d)= & {} pr_{p}\,\alpha _p\,(\theta _p-d) \end{aligned}$$

12 $$\begin{aligned} R^{*}_{(3)}(p,d)= & {} pr_{p}\,\alpha _p\,\beta _p\, (\theta _p-d) \end{aligned}$$

13 $$\begin{aligned} R^{*}_{(4)}(p,d)= & {} \gamma _p\,((\theta _p+W_p)\,\theta _p - (W_{p}+d)\,d) \end{aligned}$$

14 $$\begin{aligned} R^{*}_{(5)}(p,d)= & {} \gamma _p\,\beta _p\, ((\theta _p+W_p)\,\theta _p-(W_p+d)\,d) \end{aligned}$$

15 $$\begin{aligned} R^{*}_{(6)}(p,d)= & {} \gamma _p\, \left( \frac{(\theta _p+W_{p})\ \theta _p}{MTBT_{p} -(W_{p}+\theta _p)+1}- \frac{(W_{p}+d)\ d}{MTBT_{p}-(W_{p}+d)+1} \right) \end{aligned}$$

Now, the problem is assigning a list of patients to a set of time blocks and days with a maximizing objective function $OF^{*}_{(i)}$. Each time block can be allocated to just one specialty. Thus, the assignment problem of a list of $P_{s}$ patients belong to specialty *s* to time block (*d*, *b*) with capacity of $\tau _{(d,b)}$ can be interpreted as a single knapsack problem, where patients are items and each block is a knapsack. Indeed, for each objective function $OF^{*}_{(i)}$, the time block (*d*, *b*) is a knapsack with capacity of $C=\tau _{(d,b)}$ and each patient is an item with weight $w_{p}=L_{p}$ and value $v_{p}=R^{*}_{(i)}(p,d)$. The goal is to assign patients to time block (*d*, *b*) in order to maximize the total value of assigned patients so that the total weight of the assignment does not violate the capacity restriction. To this end, the main problem is split into $D \times B$ knapsack sub-problems. To solve each knapsack sub-problem, we implement a dynamic programming (DP) approach with memoization. The DP utilizes a recursion function to which the inputs are the number of patients, capacity of the time block, and patients weights and values. The DP is underpinned by two main concepts:

- There is a list of $n=|P_{s} |$ patients, $p \in \{1,2,\ldots ,n\}$, of specialty *s* to be assigned to time block (*d*, *b*) with capacity $C=\tau _{(d,b)}$. For each patient *p* and all possible total weights of assigned patients $w \in \{0,1,\ldots ,C\}$, the DP constructs an array $V[0\ldots n,0\ldots C]$ to store solutions, where if no patient is available, then $V[0,w]=0$ for $0\le w \le C$, and $V[p,w]=-\infty$ if $w<0$. The array *V*[*k*, *w*] keeps track of the maximum value of any subset of patients $\{1,2,\ldots ,k\}$ with total weight *w*. Hence, *V*[*n*, *C*] contains the maximum value of patients assigned to the time block.
- The DP decomposes the problem into recurring smaller sub-problems $SP_{k}$ for making decisions on the assignment of item *k* to the time block. It computes the best solution of sub-problem $SP_{k}$ as *V*[*k*, *w*] for all $0\le w \le C$ using the recursive function:
  16 $$\begin{aligned} V[k,w] ={\left\{ \begin{array}{ll} V[k-1,w] &{} \text {if } w_{k} > w\\ \max \{V[k-1,w], V[k-1,w-w_{k}]+v_{k}\} &{} \text {otherwise} \end{array}\right. } \end{aligned}$$

The recursive function returns the best possible assignment of patients to time block (*d*, *b*). To describe the DP and heuristic algorithms, we use Table 2 notations in addition to the ones we introduced previously.

**Table 2:** The DP and heuristic algorithms notations

Notation	Description
*n*	Number of patients to be planned
*C*	Block capacity
$\Lambda$	Patients weights
$\Gamma$	Patients values
$DP(\Lambda ,\Gamma ,n,C)$	The DP procedure
$\Phi [k,w]$	Assignment array by the DP procedure
$\varphi ^{*}$	Best possible assignment of patients to a time block
$\sigma _{s}$	List of all unscheduled patients of specialty *s*
${\mathcal {T}}^{s}_{(i)}$	Total value of assigning patients of $\sigma _{s}$ to day *d*
$s^{*}$	Specialty with maximum ${\mathcal {T}}^{s}_{(i)}$
$DP(s^{*})$	List of patients belong to $s^{*}$ with $Dl_{p} \ge d$

Algorithm 1 illustrates the pseudo-code of the DP procedure, which is indicated by $DP(\Lambda ,\Gamma ,n,C)$. Here, there are *n* items (patients) to be planned, vector $\Lambda$ is their weights ($w_{p}=L_{p}$) and vector $\Gamma$ shows their values, where for function *i* they are $v_{p}=R^{*}_{(i)}(p,d)$. In the DP algorithm, $\Phi [k,w]$ is the assignment array, where $\Phi [k,w]=1$ if patient *k* is assigned to a solution with total weight *w* and $\Phi [k,w]=0$ otherwise. The $\varphi ^{*}$ also indicates the best possible assignment of patients to time block (*d*, *b*).
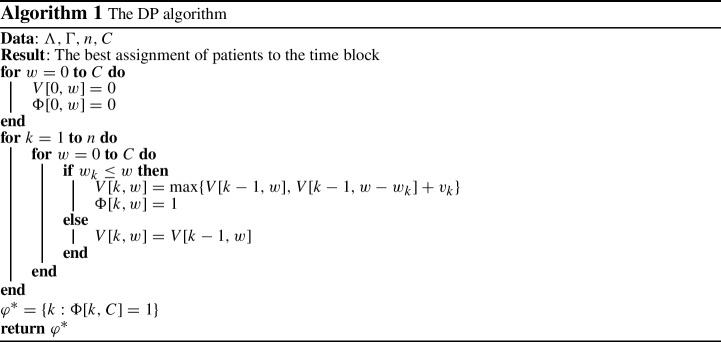


The time complexity of the DP approach for solving the Knapsack problem is of *O*(*nC*) where *n* is the number of items and *C* is the knapsack capacity. Both DPH1 and DPH2 heuristics split the problem into single knapsack sub-problems and then search for a high quality solution by solving these knapsack problems step by step and by deploying the DP algorithm. The main idea behind these algorithms is to decide which assignment of specialties and patients to a time block (*d*, *b*) lead to maximum total values assigned to ORs.

### The DPH1 algorithm

The DPH1 algorithm assumes that each time block is a knapsack. It searches for the specialty $s^{*}$ that can provide maximum total value if assigned to the time block. The assignment process begins from the first time block of the first day, i.e. block $(d,b)=(1,1)$, assuming that only this block is available for patient treatment. For each specialty *s*, the list of all unscheduled patients are listed in $\sigma _{s}$. In the next step, for each specialty *s*, the total value of assigning all patients of list $\sigma _{s}$ to day *d* is calculated as ${\mathcal {T}}^{s}_{(i)}$, where ${\mathcal {T}}^{s}_{(i)}=\sum _{p \in \sigma _{s}} \frac{R^{*}_{(i)}(p,d)}{L_{p}}$ for objective function *i*. Since the greater value of the total clinical condition deterioration for planned patients leads to having a lower objective function value in total, the owner of the block will be the specialty with the maximum value, referred as $s^{*}$.
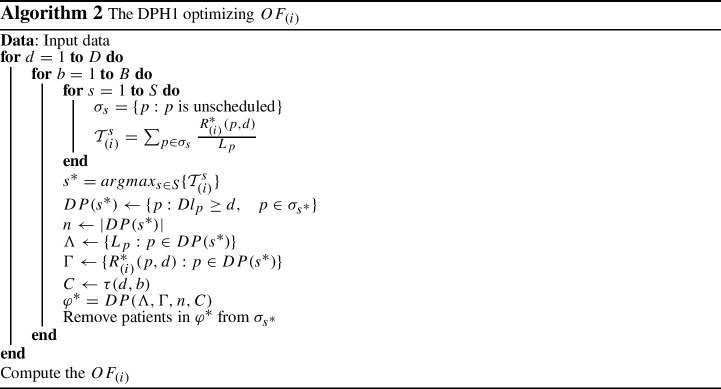


Taking into account patients due dates, the list of all patients in $\sigma _{s^{*}}$ with $Dl_{p} \ge d$, called $DP(s^{*})$, is chosen as the input of the DP algorithm. Subsequently, the DP algorithm is executed to find the best assignment of patients in list $DP(s^{*})$ to time block (*d*, *b*). Then the $\sigma _{s^{*}}$ is updated by removing all assigned patients. The algorithm will repeat all aforementioned steps for each time block to the end of the planning horizon. The whole procedure is shown in Algorithm 2. The DPH1 algorithm solves $D\times B$ knapsack problems, hence its complexity is $D\times B \times {\mathcal {O}}(nC)$.

### The DPH2 algorithm

Like DPH1, DPH2 benefits from embedded DP procedures. For every objective function $OF_{(i)}$, the DPH2 starts from the first time block in the first day, i.e. $(d,b)=(1,1)$, and treats each time block as a knapsack. For each specialty *s* with a list of unscheduled patients $\sigma _{s}$, it solves a single knapsack problem by executing the DP algorithm. Then, it computes the total value if assigning selected patients by DP to the time block as ${\mathcal {T}}^{s}_{(i)}$, where ${\mathcal {T}}^{s}_{(i)}=\sum _{p \in \varphi _{s}} \frac{R^{*}_{(i)}(p,d)}{L_{p}}$. The specialty with maximum total value, namely $s^{*}$, and associated patients selected by DP will be assigned to the time block (*d*, *b*). Thereby, the $\sigma _{s^{*}}$ will be updated by removing these patients.
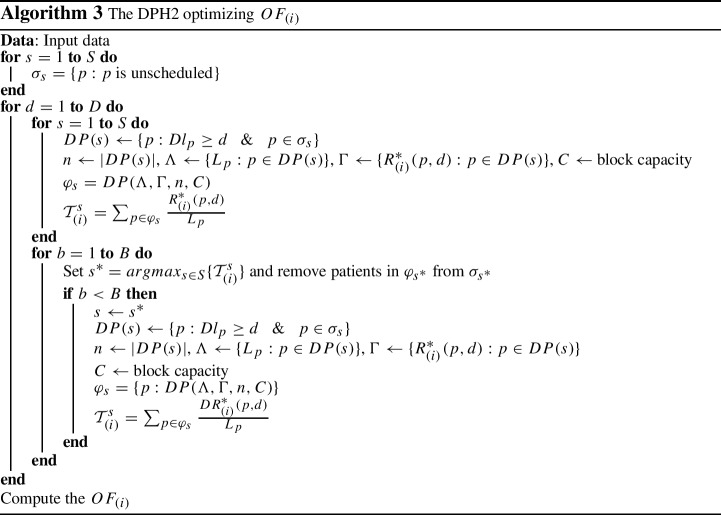


For the next time block in day *d*, the same procedure needs to be implemented. However, since for any objective function *i*, the cost does not change when assigning patient *p* to any time block *b* or $b'$ on the same day *d* due to symmetry and equity of all time blocks in a day. Hence, if $b < B$ there is no need to re-run the *DP* algorithm for other specialties except for specialty $s^{*}$ and as the result just $\sigma _{s^{*}}$ list gets updated. This speeds up the solution time by only re-solving knapsack problems when required rather than for every block and specialty. If time block (*d*, *b*) is the last time block in day *d*, i.e. $b = B$, then the procedure repeats for the next day’s available time blocks. The above steps need to be taken to the last time blocks in the planning horizon. The DPH2 algorithm, illustrated in Algorithm 3, solves *S* knapsack problems for the first block on each day and then only needs to re-solve one knapsack problem for each subsequent block. Hence, the complexity of DPH2 algorithm is ${\mathcal {D}}\times (S+B) \times O(nC)$.

## Computational experiments

The literature on the OT planning and scheduling problems is rather vast though the disclosure of the dataset is almost nonexistent thanks to patients data confidentiality. Indeed, there is almost no open access dataset that could reflect the real hospital settings and be a basis for researchers to align their methodologies, including exact and heuristic methods. To the best of our knowledge, a comprehensive benchmark dataset for the OT planning and scheduling problem does not exist in the literature. In Leeftink and Hans (2018), authors proposed a benchmark dataset for the OT planning and scheduling problems though they didn’t provide details on patients characteristics such as their waiting times, due dates, clinical priorities, etc. Hence, we propose a comprehensive and well-structured benchmark with 1080 instances motivated from the literature such as Testi et al. (2007), Fei et al. (2010), Spratt and Kozan (2016), which also provides other researchers with a dataset to perform their computational experiments. The benchmark instances are also used to thoroughly analyse our proposed approaches.

### Benchmark design

The benchmark dataset is specifically designed to investigate different characteristics of the problem and the efficiency of the proposed methodologies. It provides input values for parameters in a general OT planning and scheduling problem such as the scale of an OT department, length of planning horizon, number of surgical specialties, and patients characteristics. In this section, we discuss the procedure and reasons behind how we design these parameters’ values in the benchmark.

#### Scale of the OT department

The inherent uncertainty of surgical procedures affects the allocation of OT scarce resources to patients and the availability of ORs, and as a result the scale and size of the OT department for surgery planning. Two polices are used in allocation of ORs to emergency and elective patients, namely dedicated and flexible policies. Whilst the dedicated policy reserves some ORs for just emergency patients, flexible policy doesn’t secure any OR for emergencies and both elective and emergency patients are treated in the same ORs (VanRiet and Demeulemeester, 2015; Spratt and Kozan, 2016). Regardless of the fact that the flexible policy leads to higher overall utilization, it may result in more overtime and patients waiting times as well as less stable surgery schedules (VanRiet and Demeulemeester, 2015). In this study, we implement the dedicated policy where all ORs are considered for elective surgeries and no uncertainty contributes to the planning and scheduling process.

Moreover, some ORs may be better equipped for specific specialties and hospitals devote them to these specialties in order to decrease the cost and time of moving valuable equipment between ORs. This allocation doesn’t mean that other specialties cannot use these ORs (Spratt and Kozan, 2016). In our benchmark, all ORs have the same equipment and can be allocated to any specialty, accordingly there is no difference between time blocks of the same capacity belonging to different ORs in a day. In fact, due to the symmetry between the time blocks in each day *d*, it can be assumed that there is just one OR with several time blocks and same equipment in each day.

The number of time blocks can vary from one day to another, though for the purposes of the benchmark we assume that there are an equal number of *B* time blocks with the same capacity in each day. To determine the number of blocks in each day and their capacity, i.e. *B* and $\tau _{d,b}$, we investigated around fifty articles with real case studies. The outcome demonstrated that the number of blocks a day vary from one to fifteen blocks a day (for example see Koppka et al. (2018), Anjomshoa et al. (2018), Siqueira et al. (2018), Akbarzadeh et al. (2020), Barrera et al. (2022)). Hence, to cover real world cases, *B* is chosen from $\{4, 6, 8, 10, 12, 14\}$ and all the time blocks have the same capacity of five hours (300 min).

#### Length of planning horizon

One of the main stages in surgery planning is “admission planning" in which a surgery date is determined and the hospitalisation date is communicated with the patient (Riise and Burke, 2011). While traditionally MSS admission planning is typically one to four weeks, in recent times due to the backlog created by the COVID-19 pandemic, longer horizons have to be considered. In addition longer horizons enable additional insight into admission planning including allowing us to compare a rolling horizon approach with the global optimum over a longer period (described in the Sect. 5.2.3). Moreover, a longer MSS can assist in predicting and balancing between capacity and demand as patients clinical conditions evolve over the time. It also provides a clear picture of patients assignment to ORs and days and as a result, improved staff rostering.

As the MSS-SCAP concurrently deals with both tactical and operational levels covering short to medium term planning horizons, the number of days in the planning period is chosen from $\{5, 10, 15, 20, 40, 60\}$ days, which covers one week to twelve weeks horizons. It is notable that elective surgeries are usually planned in weekdays (i.e. Monday to Friday) and each week contains five working days.

#### Number of surgical specialties

In an OT department, there are several surgical specialties that share a fixed number of ORs. However, a limited number of them use waiting lists and their surgeries can be booked in advance (Spratt and Kozan, 2016). By delving into the literature with real-world case studies, the number of specialties (*S*) has been chosen in the range of $\{8, 12, 16\}$, which is consistent with what is done in practice.

#### Patients characteristics

In this section, we discuss patients characteristics (such as surgery durations, priority scores, and waiting times) and how we defined their values in the benchmark.

*Surgery durations*

Surgery durations are stochastic by their very nature and their variability can be addressed by having good estimates (VanRiet and Demeulemeester, 2015; Meersman and Maenhout, 2022). The uncertainty in surgery durations is acknowledged by many authors while many others disregard it in order to simplify their scheduling problems (Riise and Burke, 2011). To cope with uncertain surgery durations the most common approach is “slack planning”, where part of a block’s capacity is reserved as a buffer for unpredicted incidents and allows for deterministic planning. For example, if the available capacity is six hours, then one hour is reserved as a buffer and the rest is used for deterministic planning.

Surgeries belong to a surgical specialty are medically homogeneous and have similar treatment durations. Strum et al. showed that lognormal distribution often results in the best fit for surgical durations estimation (Strum et al., 1998). Here, the surgery durations are randomly approximated using lognormal distributions (shown in Table 3) provided by Spratt and Kozan (2016). Since the minimum time block capacity is five hours, the sampled values are truncated at 300 min to ensure each surgery fits within a block.

**Table 3:** The lognormal distributions parameters, URGs combinations, and percentage of patients per specialty

s	Name	$\mu$	$\sigma ^{2}$	$URG^{1}$	$URG^{2}$	$URG^{3}$	$URG^{4}$	$URG^{5}$	$\lambda _{s}^{S=8}$	$\lambda _{s}^{S=12}$	$\lambda _{s}^{S=16}$
1	Cardiology	0.788	0.395	0.039	0.039	0.451	0.451	0.02	1.15	0.85	0.73
2	Ear, Nose and Throat	1.2	0.513	0.059	0.059	0.371	0.371	0.141	0.44	0.33	0.28
3	General Surgery	0.788	0.395	0.109	0.109	0.289	0.289	0.204	22.68	16.75	14.39
4	Gynaecology	$-$ 0.598	0.031	0	0	0.5	0.5	0	0.05	0.04	0.03
5	Neurosurgery	0.97	0.353	0.057	0.057	0.424	0.424	0.038	5.88	4.34	3.73
6	Ophthalmology	$-$ 0.474	0.463	0.006	0.006	0.104	0.104	0.78	26.04	19.24	16.52
7	Orthopaedic	0.583	0.37	0.021	0.021	0.181	0.181	0.597	22.61	16.71	14.35
8	Plastic Surgery	$-$ 0.015	0.724	0.074	0.074	0.334	0.334	0.185	21.14	15.62	13.41
9	Urology	0.027	0.658	0.09	0.09	0.224	0.224	0.373	–	14.17	12.17
10	Vascular Surgery	0.786	0.271	0.103	0.103	0.335	0.335	0.125	–	5.08	4.37
11	Hepato-Pancreato-Biliary	0.945	0.317	0.029	0.029	0.427	0.427	0.087	–	3.42	2.94
12	Colorectal	0.783	0.534	0.029	0.029	0.427	0.427	0.087	–	3.46	2.97
13	Faciomaxillary	0.266	0.453	0.092	0.092	0.318	0.318	0.18	–	–	2.8
14	Liver Transplant	$-$ 0.031	0.196	0.005	0.005	0.084	0.084	0.822	–	–	0.05
15	Cardiac Surgical Unit	1.426	0.112	0.103	0.103	0.207	0.207	0.379	–	–	6.97
16	Upper GI and Soft Tissue	0.516	0.542	0.103	0.103	0.207	0.207	0.379	–	–	4.28

*Patients MTBTs, priority scores, and waiting times*

Following the five URGs defined in Testi and Tànfani (2008), Valente et al. (2009), namely $URG^{j}$ and $j \in \{1,2,..., 5\}$, patients are categorized into five groups where their $MTBT \in \{8, 30, 60, 180, 360\}$ (in days), respectively. Based on a patient’s MTBT, a priority score is assigned to them in such a way that a patient with higher priority has a lower MTBT, i.e. $pr_{p}\in \{45, 12, 6, 2, 1\}$. For example, if patient *p* has a $MTBT_{p}=8$, then they have a priority score of $pr_{p}=45$ and belong to $URG^{1}$, which means that the patient is of high priority and must receive their required treatment as soon as possible.

Not all of the surgeries of any specialty *s* have the same URG. Some of them has highest priority and need to be operated on early while some others can wait longer. To generate patients URGs for each specialty, we scrutinized the data provided in Spratt and Kozan (2016). They categorized patients into three groups. Therefore, to have the five urgency groups, we divided their first two groups into two other ones. Table 3 shows the lognormal distribution parameters and the URGs combinations per specialty.

For each patient *p*, the waiting time is generated based on their MTBT and sampled uniformly in $[1, MTBT_{p}]$ and their due date is also generated by deducting the patient’s waiting time from MTBT, i.e. $Dl_{p}=MTBT_{p}- W_{p}$.

*Number of patients*

Since the scale and size of the OT department impacts the number of patients that can be admitted, here we generate the number of patients as a function of available capacity over the planning horizon. In Table [2] of Spratt and Kozan (2016), we can see that some of the specialties have a higher admission rate than others. For instance, in 2012, just one surgery in gynaecology (GYN) was performed while this number for neurosurgery (NSUR) is 610 surgeries. Then, by analysis their data, we defined the percentage of patients belonging to each specialty that have been admitted on the waiting list. In Table 3, $\lambda ^{S}_{s}$ is percentage of patients for $S \in \{8, 12, 16\}$. Next, we followed a step by step procedure to generate the number of patients belonging to each specialty *s* (described in below). Using this procedure, the number of patients in the benchmark varies in the range between 64 to 2358. Finally, 10 instances with different seeds per combination of *S*, *D*, and *B* are generated, in total 1080 instances. 
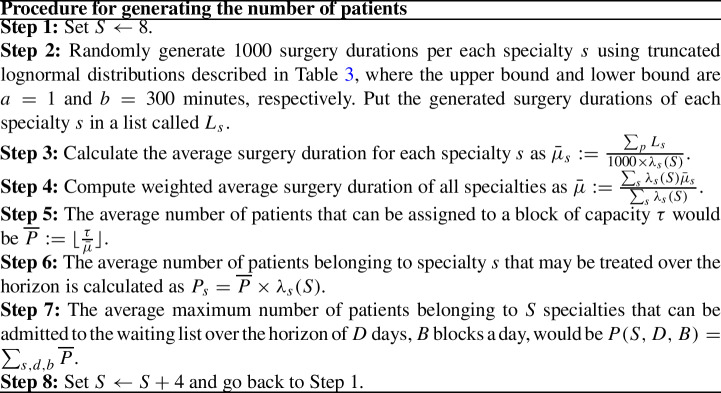


### Experimental results analysis

To investigate the performance of proposed MIP model and heuristic algorithms, we carry out a thorough computational experiments using the benchmark instances on a virtual machine equipped with Intel(R) Xeon(R) processors, @2.70GHz CPU, and 176GB RAM under the Linux operating system. The MIP models were implemented via Gurobi 9.0.1 in Python 3.7 and the heuristics are also implemented in Python 3.7. In initial experimentation, we identified that after 30 min run-time, the gap provided by Gurobi does not improve much. Hence, the MIP models were run for a maximum of 30 min.

In this section, first we discuss the solution quality and effectiveness of the six objective functions from different perspectives such as reduction in patients waiting times, increasing ORs utilization, equity and fairness of the schedules, and balancing patients and hospital administrators expectations, etc. Then, we analyse the computational efficiency of the MIP models and heuristic algorithms in terms of “optimality gap” and “run-time” across the six objective functions. At the end, we discuss solving the MSS-SCAP using “rolling horizons (RH)” approach in order to tackle the dynamic inherent of patients clinical condition and investigate the advantages/disadvantages of generating longer MSS plans.

#### Effectiveness of objective functions

**Fig. 1:**
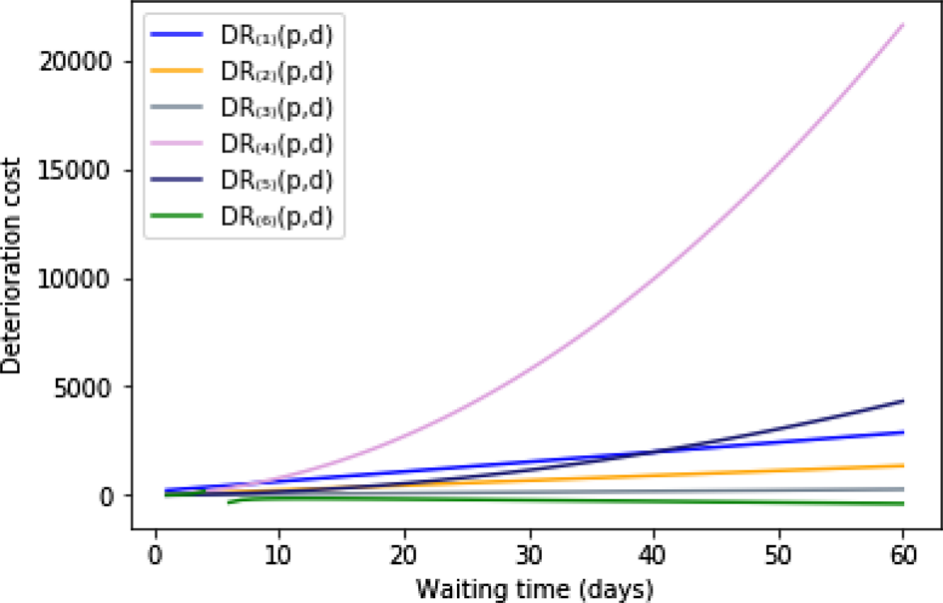
The six deterioration rates when $pr_{p}=45$, $MTBT_{p}=64$ and $W_{p}=4$

We quantify the deterioration of patients clinical condition by introducing six different deterioration rates as a function of their clinical priorities, waiting times, and due dates. Figure 1 shows the six deterioration rates for a patient with priority score $pr_{p}=45$, $W_{p}=4$, and $MTBT_{p}=64$ over a planning horizon of 60 days.

Among the six deterioration rates, objectives $OF_{(4)}$ to and $OF_{(6)}$ have a non-linear relationship with patients surgery day (total waiting times), which may result in better approximation of real-world situation, especially considering large instances. In fact, with a larger planning horizon, linear functions may not be able to provide practically viable solutions.

**Fig. 2:**
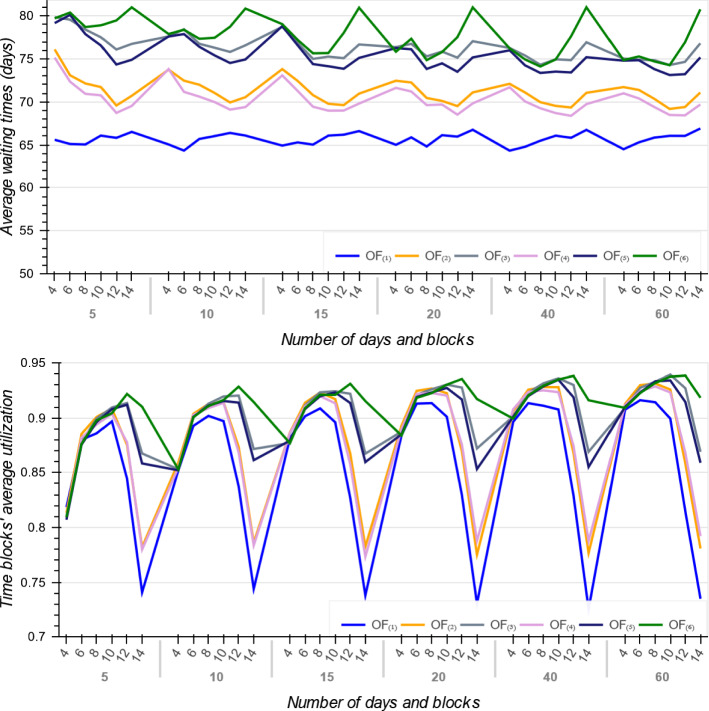
Patients average waiting times and time blocks utilization resulted by MIP models

Several factors can reflect the quality of the resulting solutions such as patients average waiting times, ORs average utilization, and the number of accepted, transferred, or rejected patients in each urgency category. Generally, patients desire to wait less and be treated more quickly. Figure 2 shows the average waiting times of accepted patients and time blocks average utilization resulted by the MIP models across the six objective functions. The waiting time here includes the time that a patient *p* already spent on the waiting list plus their waiting time on the planning horizon before their surgery day (i.e. $d+W_{p}$). We see that with the $OF_{(1)}$, accepted patients have minimum average waiting times in that the $OF_{(1)}$ is a linear function of patients surgery day, priority scores and waiting times.

As patients priorities and waiting times are input parameters, the only variable part in $OF_{(1)}$ is patients surgery day, i.e *d*. Therefore, to minimize the whole function, it is better to perform each surgery as soon as possible which equals to minimizing patients total weighted completion times. Hence, it can be concluded that $OF_{(1)}$ serves patients more quickly and does not consider if patients with higher priorities wait longer. If each patient’s surgery duration is equal to time block capacity, then $OF_{(1)}$ is the best in the sense that the total completion times of patients is minimized and the number of scheduled patients is maximized. However, it provides the least utilization rate on average (Fig. 2).

After $OF_{(1)}$, $OF_{(2)}$ and $OF_{(4)}$ have the lowest patients average waiting times and serves patients more quickly. Furthermore, they have the next least average utilization rates and don’t utilize the time blocks as good as the other three functions $i \in \{3, 5, 6\}$. Indeed, from patients point of view, the objective functions $\{3, 5, 6\}$ are not satisfactory and they are more attractive to the hospital administrators. In summary, we can say that if the objective function minimizes the patients average waiting times, then it would provide the hospital administrations with less utilization rates. For example, although $OF_{(6)}$ has the largest average utilization rate amongst all the six functions, it doesn’t provide patients with least average waiting times.

**Fig. 3:**
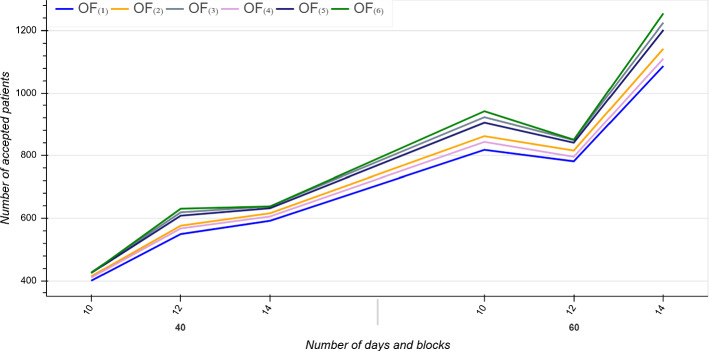
Average number of accepted patients

**Fig. 4:**
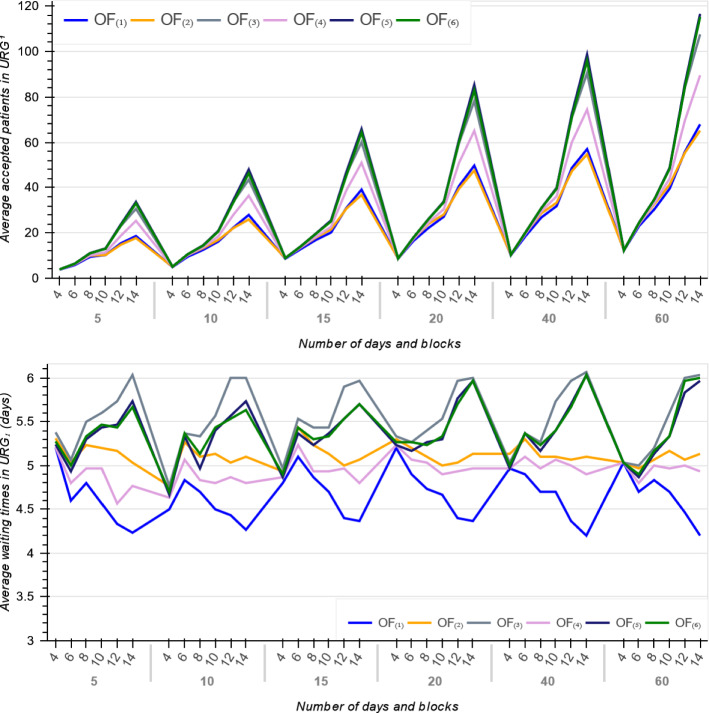
Average number of accepted patients in $URG^{1}$ and their average waiting times

Figure 3 illustrates the average number of accepted patients for large size instances with $D \in \{40, 60\}$ and $B \in \{10, 12, 14\}$ across all the objectives. As expected, $OF_{(1)}$ has the minimum average number of accepted patients. We also compared the number of accepted patients in each URG to determine which objective function focuses on patients with higher priorities. The number of accepted patients in $URG^{1}$ per objective is displayed in Fig. 4. As shown, $OF_{(1)}$ provides minimum average waiting times for patients in $URG^{1}$ but accept minimum number of patients from this urgency category. By contrast, $OF_{(3)}$ with largest average waiting times takes better care of the most urgent patients and provides better quality solutions in terms of fairness and equity.

To support above argument, we further investigate the standard deviation of the waiting times of accepted patients ($STD_{(i)}$) per each objective *i*. Within an urgency category, a larger $STD_{(i)}$ implies that the objective function *i* schedules more patients with dissimilar waiting times and thereby higher differentiate priorities. Indeed, a lower $STD_{(i)}$ (close to zero) means that patients have quite similar waiting times.

**Fig. 5:**
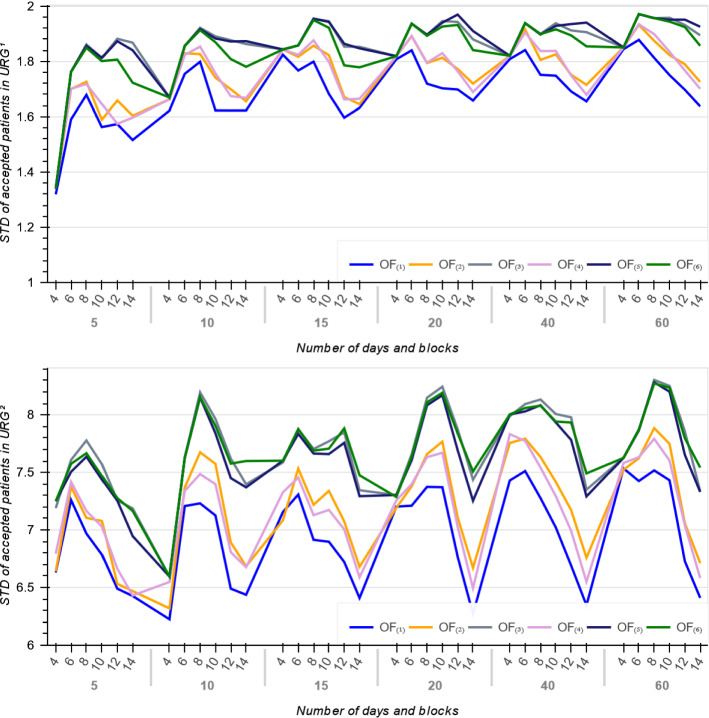
Standard deviation of accepted patients’ waiting times

Figure 5 illustrates the standard deviation of waiting times for the accepted patients in $URG^{1}$ and $URG^{2}$. As it shown, among all objectives, $OF_{(i)}$ for $i \in \{3, 5, 6\}$ have largest $STD_{(i)}$ and thus are more fair against high priority patients, but $OF_{(1)}$ with smallest *STD* for $URG^{1}$ and $URG^{2}$ is more suitable for minimizing patients average waiting times (Fig. 5). Hence, the objective functions $OF_{(i)}$ for $i \in \{3, 5, 6\}$ are more attractive to the administrators and provide more stable schedules in that they generate solutions with less stress of being close to patients due dates.

**Fig. 6:**
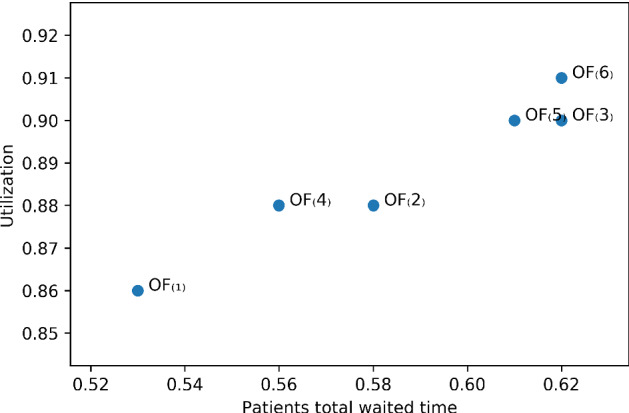
Trade-off between the blocks utilization and patients total waited time

Figure 6 shows the trade-off between two criteria including the average utilization of the blocks and accepted patients total average waiting times ($\sum _{p \in {\mathcal {A}} }\frac{W_{p}+d}{MTBT_{p}}$, where ${\mathcal {A}}$ is the list of accepted patients) across the six objective functions. We see that if the goal is to minimize patients total waiting times, then $OF_{(1)}$ is the best. The $OF_{(4)}$ provides the best trade-off between patients and administrators expectations. Moreover, if the goal is having high utilization rate, then $OF_{(6)}$ is a good option. Figure 6 also indicates that the solutions from $OF_{(2)}$ and $OF_{(3)}$ are not Pareto-optimal for the objectives of maximising utilization and minimising waiting time being dominated by $OF_{(4)}$ and $OF_{(5)}$, respectively. However, as will be shown in Sect. 5.2.3, when embedded in a rolling-horizon approach with a short look-ahead period, these dominated methods actually perform better in the dynamic environment, perhaps precisely because they do not pack the schedule too tightly in the short term. In conclusion, selecting between the objective functions depends on how the decision maker benefits, specifically, between patients or hospital administrators. From managerial perspective, $OF_{(i)}$ for $i \in \{3, 5, 6\}$ provide more stable schedules but they may result in more patients waiting times. On the other hand, if they tend to generate schedules with lower waiting times, the $OF_{(1)}$ is the best. And if a balance between patient or administrator expectations is sought, $OF_{(4)}$ and $OF_{(6)}$ are more useful, respectively.

#### Efficiency of proposed methodologies

Tables 4, 5 and 6 show the optimality gap of the MIP model, DPH1, and DPH2 all the six objective functions inclusive. The columns *S*, *D* and *B* indicate the number of specialties, number of days, and number of time blocks a day, respectively. The columns $MIP_{(G)}$ show the optimality gap of the MIP models. The final gap associated with a solve of MIP model for objective function *i* is calculated as $\frac{UB_{(i)}-LB_{(i)}}{LB_{(i)}}$, where $UB_{(i)}$ and $LB_{(i)}$ are the upper bound the lower bound for $i \in {\mathcal {O}}$.

The results demonstrate the ability of the MIP models in finding excellent solutions (maximum average gap of 4.71% across all instances and objective functions), but requires large run-times. Additionally, the $DPH1_{(G)}$ and $DPH2_{(G)}$ columns indicate the average gap of DPH1 and DPH2 to the MIP model’s lower bound, respectively. For objective function *i*, the heuristics gap are calculated as $\frac{(H^{*}_{(i)}-LB_{(i)})}{LB_{(i)}}$, where $H^{*}_{(i)}$ is the heuristic’s best solution. All the optimality gaps are reported in percentages.

**Table 4:** MIP and heuristics optimality gaps for $s=8$ across all objective functions

*D*	*B*	$MIP_{(G)}$						$DPH1_{(G)}$						$DPH2_{(G)}$					
		(1)	(2)	(3)	(4)	(5)	(6)	(1)	(2)	(3)	(4)	(5)	(6)	(1)	(2)	(3)	(4)	(5)	(6)
5	4	0	0	0	0	0	0	0.99	8.96	4.36	13.47	5.74	1.93	1.18	5.8	5.96	7.85	7.48	13.66
	6	0	0	0	0	0	0	0.91	8.21	7.61	10.19	8.59	3.71	1.33	4.27	2.56	7.47	4.08	4.69
	8	0	0.05	0	0	0	0	1.1	11.07	3.51	13.82	3.97	2.31	1.65	5.68	2.03	8.94	3.37	7.28
	10	0	0.23	0	0.3	0	0	0.7	7.4	2.5	9.1	2.96	1.56	0.9	5.34	0.97	6.61	1.16	1.67
	12	0	0.37	0.03	0.43	0	0.03	0.69	8	1.6	9.52	1.7	1.17	1.02	4.89	1.22	8.78	1.29	1.46
	14	0.02	0.4	0.04	0.54	0.04	0.03	0.73	9.48	2.54	12.14	4.18	1.9	1.26	5.98	2.28	9.67	3.22	2.61
10	4	0.01	0.16	0	0.12	0	0	2.21	30.41	19.44	39.82	43.03	19.58	2.13	7.21	4.39	9.44	9.95	20.27
	6	0.06	0.33	0.05	0.6	0.05	0.02	1.91	42.16	25.24	54.69	43.61	22.04	2.07	8.95	6.27	15.89	16.16	30.89
	8	0.09	0.77	0.14	1.11	0.14	0.13	1.88	31.05	24.95	51.85	39.96	19.19	1.83	9.96	5.07	14.4	12.25	32.66
	10	0.08	0.91	0.49	1.63	0.86	0.95	1.82	30.63	18.55	49.29	31.89	22.35	1.92	9.12	4.88	15.26	11.52	36.22
	12	0.1	1.29	0.8	2.88	1.29	1.45	2.06	30.34	18.49	46.69	26.41	20.02	1.72	9.27	4.03	14.38	10.05	40.48
	14	0.13	1.76	1.05	3.93	1.98	2.04	1.92	28.35	23.84	49.06	35.91	20.51	1.9	10	4.9	18.01	13.31	37.16
15	4	0.05	0.34	0.03	0.63	0.05	0.04	3.14	46.47	34.28	75.96	65.74	34.27	2.79	10.49	6.21	16.79	15.58	60.95
	6	0.1	0.83	0.4	1.4	0.65	0.39	2.93	43.11	43.08	84.19	54.61	51.65	2.5	11.36	9.4	18.64	15.06	62.66
	8	0.15	1.4	0.95	3.86	2.29	2.28	3.03	51.03	35.28	90.85	66.54	48.14	2.34	11.71	8.43	17.74	20.11	63.62
	10	0.2	1.89	2.36	4.56	4.24	6.59	2.97	44.78	37.81	81.89	60.38	58.76	2.5	13.02	9.78	22.71	19.44	84.85
	12	0.27	2.9	3.27	5.19	6.17	7.3	3.06	54.08	31.34	93.4	73.81	65.62	2.63	13.97	9.89	22.16	21.84	88.11
	14	0.28	3.12	3.43	5.47	5.69	7.59	2.78	44.53	31.67	88.71	63.88	61.04	2.35	14.19	8.79	21.9	17.67	68.51
20	4	0.11	0.58	0.39	1.12	0.42	0.39	3.67	52.01	49.98	106.66	85.7	77.19	2.57	10.94	12.64	21.7	25.21	91.19
	6	0.16	1.55	0.85	3.67	1.94	3.38	3.84	58.49	56.29	100.89	80.27	99.04	2.88	12.99	12.29	25.67	30.43	125.16
	8	0.29	2.56	3.03	5.16	5.58	7.87	4.38	56.03	51.01	101.55	98.16	90.54	2.91	13.93	12.03	27.71	25.84	99.66
	10	0.33	3.51	4.77	5.28	6.31	10.59	3.89	65.25	46.27	109.47	89.22	103.31	2.78	16.37	14.34	28.39	30.87	137.83
	12	0.42	3.47	3.89	8.01	6.84	11.58	3.87	54.03	41.26	115.83	83.92	101.73	2.76	15.47	13.27	27.99	25.96	122.03
	14	0.44	4.34	4.79	8.74	8.03	15.32	3.9	48.23	39.21	87.69	74.12	105.54	2.84	15.68	12.86	29.51	27.6	134.36
40	4	0.41	2.72	2	5.31	2.83	6.55	8.26	70.09	39.08	162.82	85.63	171.77	3.7	12.57	10	29.68	25.04	195.98
	6	0.62	3.62	5.98	7.49	9.39	13.93	7.14	79.76	46.85	157.18	102.94	202.71	3.62	14.97	14.66	33.22	28.87	232.28
	8	0.62	3.71	6.38	9.66	9.85	19.11	6.58	77.53	44.84	161.09	97.05	216.82	3.5	15.15	14.18	34.68	31.32	243.84
	10	0.54	5.47	6.06	5.69	9.77	18.87	6.91	76.38	44.01	158.24	101.59	222.93	3.45	15.93	14.37	37.73	31.61	272.07
	12	0.98	6.12	6.06	6.47	9.29	20.72	7.11	72.17	37.87	144.97	93.49	228.16	3.33	14.64	14.54	33.67	30.5	260.23
	14	1.15	6.54	6.6	6.11	10.41	23.13	6.25	79.41	41.15	154.8	99.11	229.65	3.28	14.82	14.58	36.32	31.82	269.93
60	4	0.63	3.44	3.62	6.89	5.17	9.06	10.34	75.08	35.11	145.36	69.8	220.39	4.02	13.51	9.83	31.86	22.14	249.41
	6	0.75	4.17	5.66	8.71	8.03	19.85	10.52	74.87	35.12	160.02	85.32	266.14	3.7	13.83	12.36	34.31	27.59	315.79
	8	0.79	5.31	5.59	10.72	9.46	24.17	9.36	76.89	35.07	148.47	91.85	291.66	3.77	14.86	11.68	34.74	30.2	335.35
	10	1.3	5.69	5.75	7.97	11.95	27.51	8.64	74.97	33.07	156.49	81.5	259.79	3.91	14.54	11.79	36.14	27.64	309.05
	12	1.92	5.1	5.79	7.43	10.9	30.03	9.7	79.57	34.59	151.82	88.45	268.25	3.72	15.02	12.08	35.86	27.28	338.87
	14	1.84	4.91	5.91	8.31	9.94	31.14	9.25	77.36	34.45	164.58	83.4	274.3	3.74	15.05	11.84	37.24	27.48	331.54

**Table 5:** MIP and heuristics optimality gaps for $s=12$ across objective functions

*D*	*B*	$MIP_{(G)}$						$DPH1_{(G)}$						$DPH2_{(G)}$					
		(1)	(2)	(3)	(4)	(5)	(6)	(1)	(2)	(3)	(4)	(5)	(6)	(1)	(2)	(3)	(4)	(5)	(6)
5	4	0	0	0	0	0	0	1.94	10.38	3.17	16.79	4.04	7.05	2.31	7.63	5.33	10.89	5.88	12.84
	6	0	0	0	0	0	0	1.34	14.44	3.85	17.44	18.83	3.4	1.45	5.93	1.77	8.54	6.71	8.59
	8	0	0	0	0	0	0	1.43	11.65	6.83	21.69	5.59	2.51	1.78	4.93	2.1	7.44	2.92	5.62
	10	0	0.08	0	0.28	0	0	1.13	13.09	1.89	13.48	2.37	1.79	1.34	5.01	1.05	8.55	3.85	4.72
	12	0	0.24	0.01	0.3	0	0	1	15.77	6.22	14.42	5.95	3.17	1.19	5.93	1.3	8.43	2.85	3.3
	14	0.01	0.48	0.06	0.68	0.02	0.03	1.05	17.43	2.52	18.54	2.65	1.54	1.26	5.21	1.42	9.93	1.8	1.82
10	4	0.06	0.06	0	0.19	0	0	2.25	41.96	30.94	57.01	37.52	18.24	1.9	6.61	5.06	9.04	7.69	14.52
	6	0.05	0.47	0.01	0.67	0.01	0.03	3	46.54	27.77	76.6	35.92	21.13	2.45	9.16	5.09	14.42	8.31	32.39
	8	0.09	0.61	0.08	0.91	0.1	0.15	2.72	48.32	22.61	70.24	35.11	20.88	2.22	8.65	5.8	13.31	8.93	26.17
	10	0.1	1.01	0.42	2.55	0.68	0.88	2.23	43.11	30.43	59.36	37.61	21.49	1.9	8.65	4.99	14.26	10.67	24
	12	0.16	1.39	0.84	3.81	2.12	3.13	2.68	50.44	30.91	84.05	45.62	23.11	1.92	8.96	5.11	16.45	12.76	36.49
	14	0.34	2.22	1.58	5.52	3.61	3.88	2.64	47.63	24.62	82.94	41.61	18.51	2.12	9.1	4.34	15.56	16.49	35.92
15	4	0.1	0.74	0.04	0.83	0.08	0.03	4.21	57.46	56.39	94.55	77.27	29.16	2.65	10.04	7.57	14.82	12.94	43.36
	6	0.23	1.45	0.44	2.61	0.72	0.84	4.13	59.14	50.68	91.76	78.8	43.77	2.38	8.85	6	14.56	13.14	50.4
	8	0.43	2.46	1.09	4.86	2.93	3.11	4.17	62.52	36.9	122.76	82.13	50.61	2.64	11.05	6.97	18.02	15.45	55.42
	10	0.54	3.77	4.11	6.31	5.99	9.2	4.48	57.31	48.01	108.61	87.91	56.5	2.66	12.88	9.43	21.09	19.8	68.21
	12	0.58	4.88	6.24	6.27	7.8	13.2	4.33	55.48	63.46	101.49	100.99	71.1	2.72	13.47	10.88	20.46	21.19	76.7
	14	0.55	4.76	6.01	6.6	8.17	14.63	4.19	58.92	46.35	101.61	71.23	65.01	2.37	13.09	9.46	20.72	18.7	65.63
20	4	0.18	0.99	0.2	1.27	0.19	0.26	5.43	57.09	44.41	113.9	80.86	69.93	3.16	10.38	8.68	18.28	15.84	69.27
	6	0.45	2.52	2.51	4.43	3.96	6.31	5.74	65.02	44.46	121.03	75.59	78.54	3.08	11.43	9.72	20.27	19.18	89.54
	8	0.6	4.27	5.3	5.13	7.5	14.28	5.3	73.36	55.86	136.73	103.14	91.07	2.73	13.15	11.54	21.17	20.91	106.46
	10	0.6	4.37	6.23	8.41	8.69	12.51	5.39	71.31	59.53	117.19	99.2	99.83	2.87	12.8	12.57	24.79	24.49	117.62
	12	0.83	4.85	7.56	10.91	12.1	17.7	5.68	69.22	54.77	132.31	111.88	125.01	3.05	14.14	13.64	27.99	27.95	130.9
	14	1.03	4.96	6	10.52	12.09	22.33	5.42	67.71	62.37	135.34	104.18	104.44	2.84	14.24	13.1	25.53	25.61	124.45
40	4	0.82	3.08	2.83	7.12	3.71	6.7	9.73	84.63	49.33	164.11	97.93	169.91	3.78	11.41	10.64	28.96	20.95	187.81
	6	0.98	4.94	8.34	10.01	9.56	22.8	9.38	79.61	47.82	164.78	105.19	185.58	3.42	13.04	13.07	29.01	23.31	190.54
	8	1.32	5.18	9.2	15.79	11.46	24.68	9.27	84.99	51.2	174.97	104.44	213.54	3.5	13.88	13.13	30.3	27.73	224.52
	10	0.7	5.65	6.98	6.7	12.69	24.76	9.25	80	52.15	151.75	103.98	216.83	3.66	14.22	12.82	31.51	26.4	215.89
	12	1.15	6.1	8.44	7.79	13.32	29.23	8.88	79.78	47.91	166.11	94.51	231.98	3.46	13.86	13.55	32.72	28.92	233.6
	14	0.99	7.02	7.16	7.32	12.95	30.8	8.64	77.52	46.45	153.91	95.85	221.87	3.3	14.33	13.09	30.55	28.22	227.45
60	4	1.35	3.7	5.72	9.8	5.54	11.42	11.28	80.91	42.64	160.71	87.9	207.72	4.13	12.41	8.96	29.38	19.85	218.01
	6	1.33	4.98	7.61	10.23	11.66	24.62	11.98	80.59	42.98	165.66	92.75	256.28	3.83	13.46	11.57	30.34	24.78	272.27
	8	1.28	5.8	7.64	13.1	12.27	26.49	11.44	74.61	41.66	161.09	96.41	254.22	3.93	13.94	10.22	30.39	24.33	274.31
	10	1.51	7.06	7.15	7.67	13.25	33.22	12.13	76.01	42.76	170.44	87.8	266.14	3.77	13.94	10.71	31.86	24.79	289.08
	12	1.87	7.17	7.04	8.61	12.86	35.02	11.54	75.26	37.69	156.36	92.97	256.46	3.73	13.91	10.91	31.55	26.46	281.07
	14	1.89	7.53	7.98	10.43	11.33	34.74	11.23	78.06	39.06	160.69	95.22	262.53	3.78	14.03	10.76	32.56	25.65	289.72

**Table 6:** MIP and heuristics’ optimality gaps for $s=16$ across objective functions

*D*	*B*	$MIP_{(G)}$						$DPH1_{(G)}$						$DPH2_{(G)}$					
		(1)	(2)	(3)	(4)	(5)	(6)	(1)	(2)	(3)	(4)	(5)	(6)	(1)	(2)	(3)	(4)	(5)	(6)
5	4	0	0	0	0	0	0	2.62	17.82	10.18	29.56	15.5	7.75	2.67	9.65	4.05	17.92	6.91	10.88
	6	0	0	0	0	0	0	2.28	22.75	2.04	30.86	2.62	5.26	2.06	5.8	3.22	9.13	5.74	7.62
	8	0	0	0	0.01	0	0	1.79	20.72	7.48	24.24	8.12	6.99	1.98	6.19	3.36	11.02	7.15	11.41
	10	0	0.16	0	0.11	0	0	1.5	18.81	6.61	17.93	10.54	4.96	1.68	6.46	1.72	9.43	7.39	14.04
	12	0	0.45	0	0.15	0	0	2.03	28.44	5.92	36.67	7.4	3.3	1.85	7.88	1.92	11.45	7.51	14.14
	14	0.01	0.72	0.05	0.39	0.03	0.02	1.59	12.59	4.2	18.66	11.53	2.96	1.52	7.36	1.45	8.68	6.41	9.71
10	4	0.04	0.16	0	0.12	0	0	3.76	46.09	42.35	64.52	72.06	25.18	2.86	7.11	4.07	10.83	9.33	25.81
	6	0.08	0.38	0	0.32	0.01	0	3.27	43.66	22.36	53.84	45.54	25.49	2.31	8.76	6.66	12.77	13.36	31.62
	8	0.1	1.29	0.12	1.03	0.15	0.09	3.33	47.85	40.93	71.12	54.07	24.78	2.47	8.93	6.69	13.4	11.42	28.76
	10	0.14	1.68	0.51	0.95	0.71	0.47	2.84	52.77	43.04	80.25	67.35	25.19	2.31	9.73	6.43	13.03	11.66	38.94
	12	0.27	2.53	1.33	3.47	2.85	1.23	3.28	49.55	38.7	76.04	75.81	25.55	2.13	10.05	4.5	15.84	14.78	29.69
	14	0.41	3.63	3.7	5.93	5.21	3.55	3.19	47.32	28.62	81.72	73.63	33.6	2.63	11.4	7.69	18.4	20.61	38.57
15	4	0.12	0.49	0.03	0.49	0.04	0.02	5.32	59.68	46.28	104.75	83.3	40.37	3.09	7.7	5.85	14.31	10.11	39.5
	6	0.19	1.32	0.48	1.87	0.32	0.46	4.83	43.37	41.49	91.11	82.13	43.42	2.91	10.19	7.68	16.95	12.36	45.81
	8	0.32	2.51	1.37	3.3	1.65	1.56	5.08	66.68	41.13	113.19	73.39	49.9	2.78	10.51	7.35	18.49	15.08	59.8
	10	0.57	4.5	3.61	5.71	5.65	5.02	5.36	66.81	57.31	120.13	107.49	54.98	3	13.76	10.52	22.6	24.13	67.72
	12	0.62	4.59	5.99	8.12	8.44	9.88	4.81	68.29	55.72	115.39	94.1	54.46	2.69	12.37	10.96	21.36	22.14	75.45
	14	0.79	5.56	7.06	7.83	9.24	15.24	4.81	53.48	56.24	106.23	101.27	66.12	2.75	12.11	11.04	20.62	22.31	75.7
20	4	0.16	1.13	0.38	1.31	0.25	0.4	5.66	56.94	53.42	127.67	80.58	60.87	2.85	8.57	9.53	15.15	11.77	50.7
	6	0.35	2.93	1.79	3.65	1.66	2.91	6.16	71.15	50.39	123.26	100.62	75.66	3.15	9.89	9.51	18.77	17.97	79.54
	8	0.58	5.96	8.26	8.06	7.92	15.67	6.57	68	63.67	122.76	120.9	97.78	3.29	13.13	14.06	25.84	23.1	92.24
	10	0.84	7.01	8.01	8.25	11.2	18	6.38	61.55	59.59	125.56	121.33	111.16	3.24	12.63	14.86	24.04	27.06	101.79
	12	0.95	7.16	8.21	8.11	12.32	18.88	6.35	63.98	58.81	123.52	121.74	121.08	3.13	12.27	14.75	26.37	29.41	125.4
	14	0.98	8.05	7.03	9.34	11.66	17.63	6.5	73.78	55.64	127.96	112.15	102.1	2.92	12.27	14.2	24.25	26.2	109.88
40	4	0.69	3.72	3.29	7.66	2.79	6.78	10.4	79.55	47.33	148.7	94.85	142.94	3.9	10.7	8.66	25.63	16.06	131.47
	6	1.05	4.81	9.23	11.34	11.06	22.48	10.46	66.7	54.4	144.26	108.16	180.46	3.74	11.82	13.2	27.48	24.21	179.31
	8	1.06	5.81	8.51	12.63	12.3	25.43	11.02	87.62	46.58	154.78	102.78	195.63	3.56	12.22	12.69	27.21	23.41	182.32
	10	1.16	5.44	7.4	7.14	12.71	23.6	11.06	80.3	51.09	154.5	101.87	203.34	3.48	12.46	11.74	29.44	24.98	192.81
	12	1.1	6.01	8.14	8.78	13.41	25.48	10.52	76.55	51.23	153.39	100.05	190.69	3.53	12.91	12.12	29.8	24.58	194.13
	14	1.39	7.82	8.21	9.37	12.13	30.57	10.4	74.65	44.98	153.59	92.99	202.04	3.42	12.68	11.81	29.36	24.9	208.91
60	4	0.95	3.85	4.1	9.62	8.27	11.61	13.05	76.27	37.72	150.24	82.44	169.84	3.73	10.89	7.62	24.49	18.86	158.92
	6	1.08	4.83	7.81	10.47	11.49	26.3	12.63	77.69	43.17	156.53	86.19	208.1	3.77	12.23	10.02	26.74	21.28	219.57
	8	1.41	5.57	7.75	11.14	12.19	28.84	13.24	77.63	36.29	158.73	84.23	219.84	3.69	12.35	9.66	27.96	22.26	225.71
	10	1.69	6.6	7.24	9.53	12.29	30.72	13.27	73.2	37.12	147.4	82.9	219.77	3.87	12.23	9.65	29.18	21.57	234.64
	12	1.93	7.21	8.38	9.77	12.98	30.63	12.74	74.56	35.21	151.34	85.5	221.52	3.72	12.5	9.39	28.57	20.82	234.19
	14	2.05	7.52	8.3	9.63	13.35	29.23	12.15	80.97	41.3	156.56	84.38	226.53	3.72	12.75	9.78	29.65	21.66	246.46

**Fig. 7:**
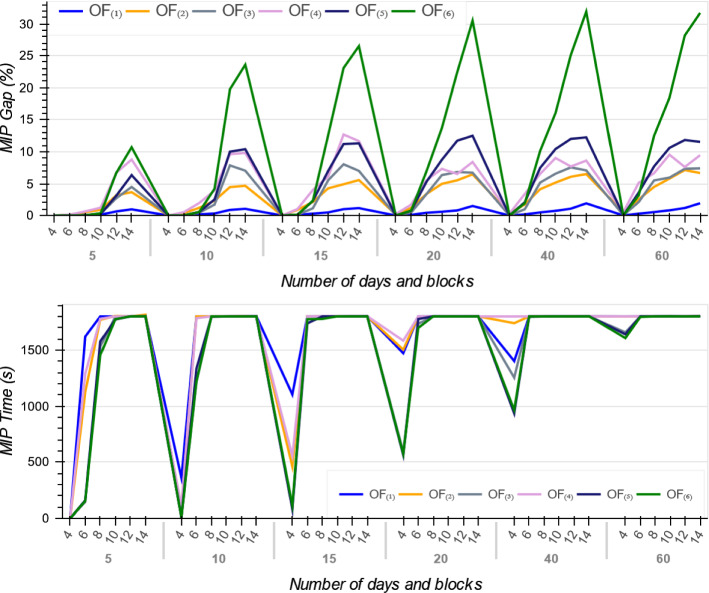
The MIP gap and run-time across all six objective functions

As reported in Tables 4, 5 and 6, for all the six objective functions and across all instances, the MIP models gap increase when the number of time blocks and days increase. It means that increasing the OT size requires more run-time for the MIP models (see Fig. 7). The reason is because a larger number of time blocks and days requires the solver to create a large number of branches to ensure the best assignment of patients to every time block is achieved, thereby requiring a much larger computational effort and declining in the MIPs performance.

Figure 7 illustrates the trends on MIPs average optimality gap and their run-time in terms of the OT planning problem scale, i.e. number of days and blocks over the planning horizon. As it shown, while $OF_{(1)}$ has the smallest MIP average gap, the average gap for $OF_{(6)}$ drastically increases when the OT size grows. The $OF_{(1)}$ has a linear relationship with patients surgery day *d* and thus in 30 min, solutions with this objective are found with lower gaps than the other objective functions, especially $OF_{(6)}$.

**Fig. 8:**
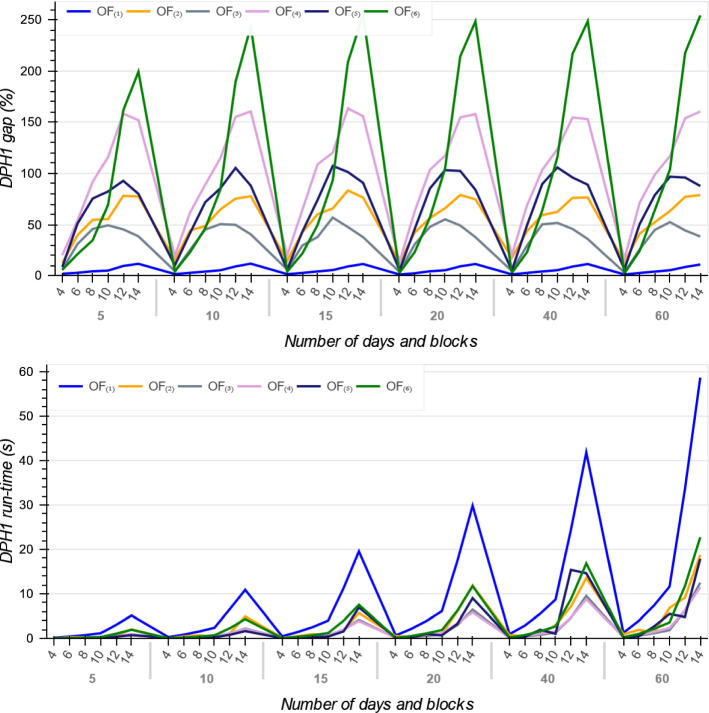
The DPH1 gap and run-time

**Fig. 9:**
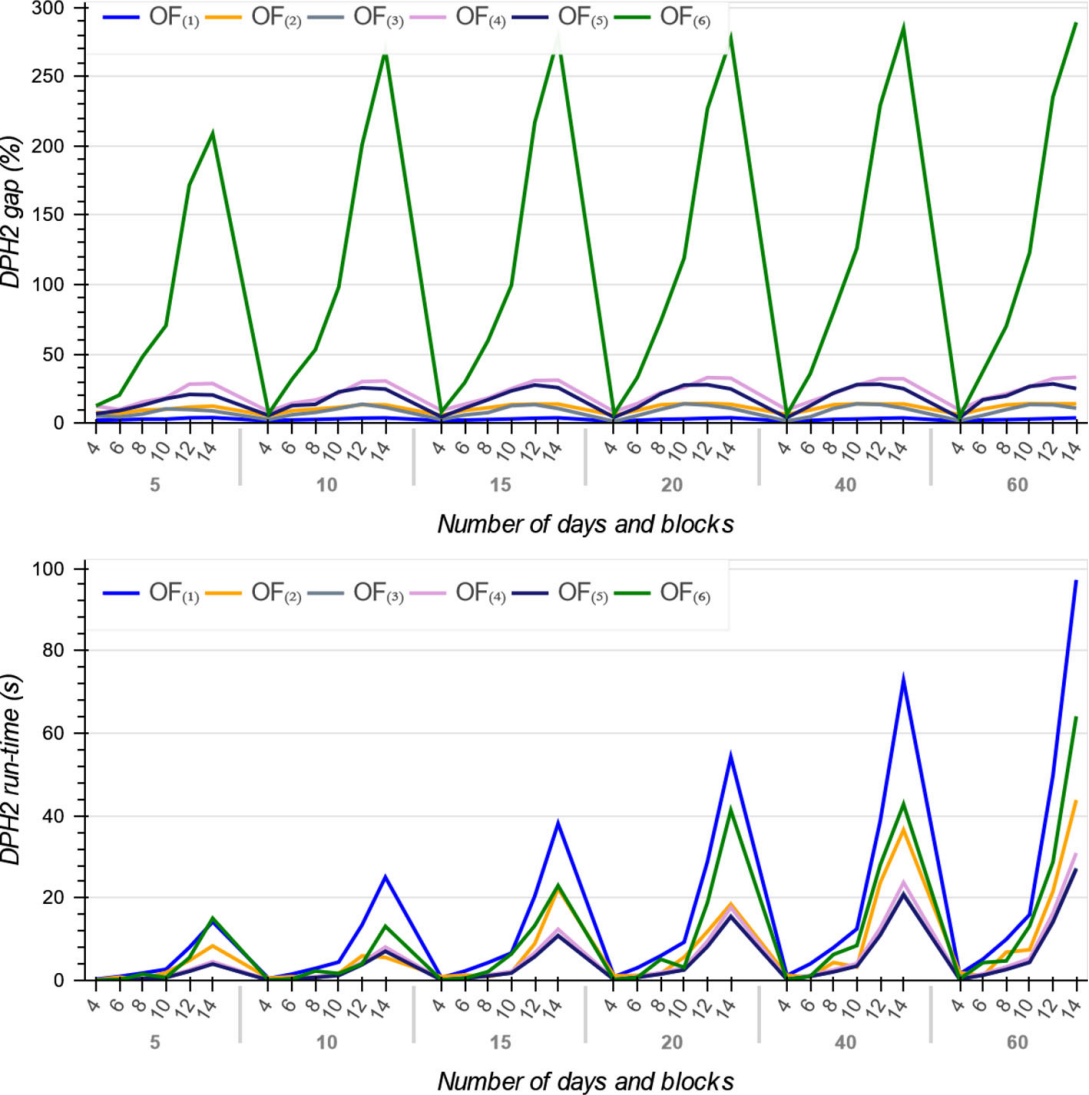
The DPH2 gap and run-times

From Tables 4, 5 and 6, we can also conclude that the number of specialties does not have a significant effect on any of the methods in terms of run-time or optimality gap. The same pattern appears in the run-time and gaps of the DPH1 and DPH2 algorithms for all the six objective functions. Figure 8 highlights a distinct advantage provided by the DPH1 algorithm, which solves all problem instances in less than 60 s. However, the DPH2 algorithm dominates the DPH1 algorithm by providing less than 25% optimality gap for all objective functions except for $OF_{(6)}$ as seen in Fig. 9.

#### Rolling horizon approach

We also solve the MSS-SCAP using a rolling horizon approach to see how the schedules can benefit from a longer MSS plan. In this approach, we solve a number of smaller MIPs by only considering certain time points within the original MIP. In other words, our time-dependent MIP model is solved repeatedly by shifting the planning intervals forward in time until the end of the horizon. Specifically, starting from the first day, the MIP model is solved for $N_{RH}$ intervals consisting of length ${\mathcal {L}}$. At the end of each rolling interval, the first *R* days scheduling is fixed and new patients arrive. Hence, the subsequent interval has an overlap of ${\mathcal {L}}- R$ days with the current one. Essentially, the planning horizon of *D* days is divided into [*D*/*R*] overlapping intervals, each shifted *R* days forward.

Using the RH approach to solve the MSS-SCAP can be beneficial from two points of view: (i) although the RH solution is not guaranteed to be optimal, it enables us to compare the solution quality between a long MSS and repeating a short-length MSS over the planning horizon; and (ii) the waiting list changes over time and more high priority patients will arrive, where the RH allows admission of those patients.
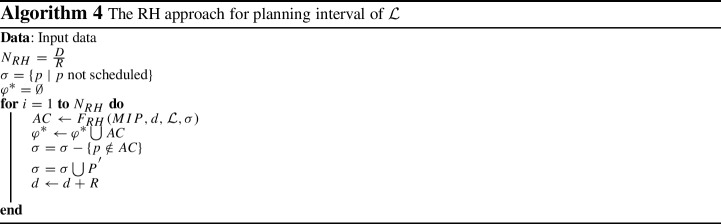


Algorithm 4 outlines the main steps of the RH approach for the objective function $OF_{(i)}$ with rolling interval ${\mathcal {L}}$. In this procedure, the $F_{RH}(MIP, d,{\mathcal {L}},\sigma )$ is a function indicating the list of accepted patients by solving the MIP model for a list of $\sigma$ patients over a planning horizon of ${\mathcal {L}}$ days starting from day *d*. In addition, $P^{'}$ is the list of new arrived patients (with $W_{p}=0$), where $P^{'}$ is a function of the available OT capacity for one week.

**Fig. 10:**
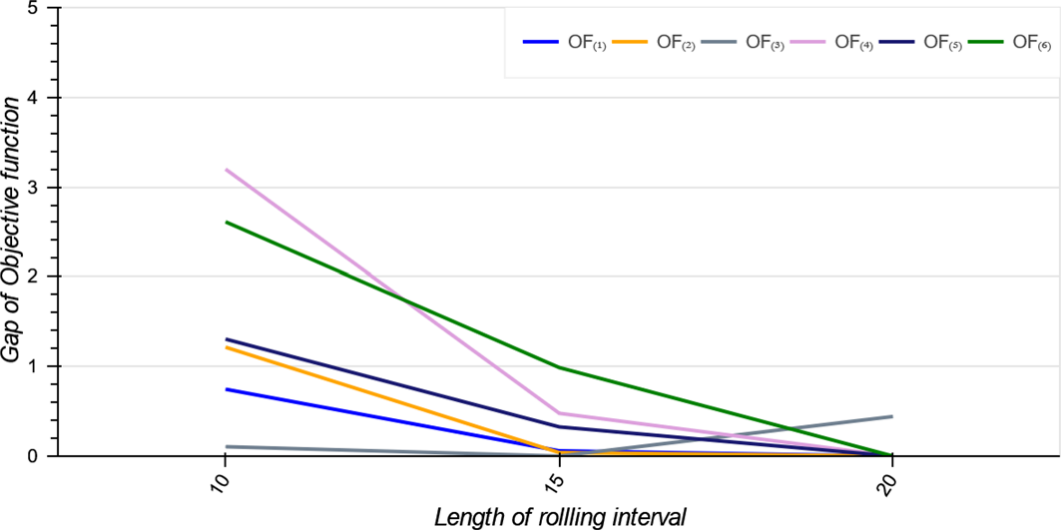
The gap of the objective functions to the minimum value

We solve problem instances with maximum number of specialties and days, $S=16$ specialties and $D=60$ days with $B=4$ blocks a day. The instances are solved for rolling intervals of ${\mathcal {L}} \in \{10,15,20\}$ and in each rolling interval, the MIP is allowed 900 s of wall-clock time. Figure 10 shows the average gap of the objective functions, where for objective function *i* the gap is calculated as $\frac{(OF^{*}_{(i)}-min_{OF^{*}_{(i)}})}{min_{OF^{*}_{(i)}}}$ in percentage. We see that when we generate a longer MSS and look further ahead, we generate schedules with lower penalties in terms of patients total clinical condition deterioration across all the six objective functions. The increase for a rolling length of 20 with objective function $OF_{(3)}$ is an artefact of the inability of the solver to get an optimal solution within the time limit.

## Conclusion

This paper deals with the synchronizing problem of both tactical and operational planning in operating theatres, namely MSS-SCAP, and presents six new objective functions. Each of the six objectives measures the total deterioration in patients clinical condition through a function (linear or non-linear) of their clinical priorities, waiting times, and due dates. To allocate blocks of time to surgical specialties, assign each patient a day for surgery, and minimize the objective functions, a general MIP model and two dynamic programming based heuristic algorithms are proposed. The MIP models provide a framework to quantify the solution quality and examine each objective function’s capability in satisfying different expectations considering all stakeholders. For the first time we propose a novel and well-designed benchmark with 1080 real-life based instances, inspired by previous studies, which provide researchers with a ideal platform to carry out their own computational experiments.

We have conducted numerical studies on the benchmark instances, which examine how the proposed model scales. The main outcome is that a larger number of days and time blocks in a day will lead to bigger optimality gaps and run-times, while the number of specialties does not seem to be a factor that has an affect. In addition, the results accurately reflect different impacts of choosing the objective function on the quality of solutions, capturing the most important aspects of the OT planning. We also analysed the solutions in terms of utilization rate across all the six objective functions and realize that the objectives $OF_{(i)}$ ($i \in \{3,5,6\}$) get the most satisfaction of the available capacity of operating rooms. The objectives also take care of most urgent patients by accepting as many as them that possible in a timely fashion. However, $OF_{(1)}$ is outstanding in terms of yielding minimum patients average waiting times and serves patients faster. Finally, $OF_{(2)}$ and $OF_{(4)}$ are the best if the goal is to balance hospital productivity and quality of service.

Future work will extend current models by taking the uncertainty of surgical cases into account. In addition, the proposed RH approach takes just the arrival of elective patients into account. However, the current patients condition and their priorities changes over the time and need to be re-assessed. Thereby, another direction can include weekly planning, where patients clinical priorities are re-assessed in a master problem that is solved over a rolling horizon of 7-days. Moreover, further research can be conducted to develop other exact and incomplete solution approaches, for example, employing decomposition methods such as Dantzig–Wolfe decomposition and/or column generation approaches. Although the proposed heuristic algorithms provide a feasible solution in less than 60 s for the six objective functions, their corresponding optimality gaps are still large and in some cases can be improved significantly, especially for $OF_{(6)}$. Therefore, from a methodological point of view, the proposed heuristic approaches can be further extended to be more sophisticated, for example, by combining them with mathematical models leading to matheuritics.
